# A Mechanism Leading to Changes in Copy Number Variations Affected by Transcriptional Level Might Be Involved in Evolution, Embryonic Development, Senescence, and Oncogenesis Mediated by Retrotransposons

**DOI:** 10.3389/fcell.2021.618113

**Published:** 2021-02-11

**Authors:** Yunpeng Sui, Shuanghong Peng

**Affiliations:** ^1^Department of Functional Neurosurgery, Beijing Neurosurgical Institute, Capital Medical University, Beijing, China; ^2^Independent Researcher, Beijing, China

**Keywords:** copy number variation, transcription, evolution, embryonic development, senescence, oncogenesis, homologous recombination, retrotransposons

## Abstract

In recent years, more and more evidence has emerged showing that changes in copy number variations (CNVs) correlated with the transcriptional level can be found during evolution, embryonic development, and oncogenesis. However, the underlying mechanisms remain largely unknown. The success of the induced pluripotent stem cell suggests that genome changes could bring about transformations in protein expression and cell status; conversely, genome alterations generated during embryonic development and senescence might also be the result of genome changes. With rapid developments in science and technology, evidence of changes in the genome affected by transcriptional level has gradually been revealed, and a rational and concrete explanation is needed. Given the preference of the HIV-1 genome to insert into transposons of genes with high transcriptional levels, we propose a mechanism based on retrotransposons facilitated by specific pre-mRNA splicing style and homologous recombination (HR) to explain changes in CNVs in the genome. This mechanism is similar to that of the group II intron that originated much earlier. Under this proposed mechanism, CNVs on genome are dynamically and spontaneously extended in a manner that is positively correlated with transcriptional level or contract as the cell divides during evolution, embryonic development, senescence, and oncogenesis, propelling alterations in them. Besides, this mechanism explains several critical puzzles in these processes. From evidence collected to date, it can be deduced that the message contained in genome is not just three-dimensional but will become four-dimensional, carrying more genetic information.

## Introduction

Copy number variations (CNVs) are mutations that occupy a large part of the genome (Henrichsen et al., 2009), and retrotransposons, such as Alu elements and long interspersed nuclear element-1 (LINE-1) constitute a huge proportion of the genome as well. It is thus unreasonable to regard them as less important or even as just junk in the long process of evolution. Whether a relationship exists between them, and if so what kind, is an intriguing problem worth exploring. LINE-1 can be inserted into the genome of the neuronal precursor cell, increasing gene expression, and influencing differentiation (Muotri et al., 2005), which indicates that the genome might not remain static during biological processes. In addition, the fact that any mRNA can be reverse-transcribed and retrotransposed with the help of open reading frame 1 protein and open reading frame 2 protein suggests that the genome can be changed by retrotransposons according to transcription (Esnault et al., 2000; Dewannieux et al., 2003). Moreover, change in CNVs is directly observable during embryonic development (Zhu et al., 2018), which demonstrates that genome alteration might be required for fundamental activity. Furthermore, the strong positive correlation between CNVs and the expression of the majority of genes (Shao et al., 2019) suggests that CNVs play an important role in determining expression. Ample evidence from neurodegenerative diseases shows that the sequence of genes changes with age and is influenced by expression (Fortune et al., 2000; Cleary et al., 2010; Lee et al., 2010; Field et al., 2019). Taken together, these findings suggest that the genome might be continuously changed by retrotransposons according to the transcriptional level of the corresponding genes throughout the whole process from fertilization to senescence, resulting in alterations and potentially even cancer. In this article, we propose that as the cell divides retrotransposons continuously and spontaneously extend the CNVs of genes depending on the genes' transcriptional level and that these changes might be involved in evolution, embryonic development, senescence, and oncogenesis.

## Indications from Neurodegenerative Diseases

The occurrence of Huntington's disease (HD) correlates closely with trinucleotide CAG repeats in the *IT15* gene. But the number of CAG repeats is unstable during the life of the HD patient and frequently even varies among generations. The instability of the CAG repeats is related to age and is tissue specific, in that tissues with higher corresponding gene expression gain more repeats than other tissues (Fortune et al., 2000; Cleary et al., 2010; Lee et al., 2010). Furthermore, a case report of a disease correlating with the abnormal expansion of CGG repeats between 55 and 200 in the fragile X retardation 1 (*FMR1*) gene called fragile X-associated tremor/ataxia syndrome showed that tissues with less methylated repeats and elevated expression of *FMR1* had increased CGG repeats (Field et al., 2019). The relationship between higher expression and increased repeats has also been observed in animal experiments. In one study, CAG repeats increased much faster in the central nervous system than in peripheral tissues of HD model mice (Vatsavayai et al., 2007) in a manner positively correlated with *IT15* expression (Li et al., 1993). It is interesting that B6CBA-Tg(HDexon1)62Gpb/3J, a strain of transgenic HD model mice, exhibits a higher incidence of CAG repeat expansion when the transgene is transmitted via paternal inheritance (according to the Jackson Laboratory), which might be a result of the wide overexpression of genes in sperm (Soumillon et al., 2013).

Besides expression, the insertion or contraction of repeats is also associated with methylation. Fragile X syndrome (FXS) is a disease associated with abnormal expansion of more than 200 CGG repeats in the *FMR1* gene. In this disease, which results in intellectual disability and autism, expression of the *FMR1* gene is greatly inhibited due to hypermethylation (Bell et al., 1991). It was reported that CGG repeats contracted to more than 160 repeats in the testes of an FXS fetus with lower methylation, whereas repeats in the brain remained over 200 with higher methylation (Duan et al., 2016). In another report, the brain of a male FXS patient with a hypermethylated *FMR1* gene barely expressing the *FMR1* protein showed full mutation of *FMR1*, whereas the testis with less methylation exhibited a premutation of 60 CGG repeats. In this experiment, a lung tumor of the patient with FXS was also tested, showing contracted CGG repeats to a premutation of 160 repeats. Expression of FMR1 protein detected in the lung tumor also indicated decreased methylation (de Graaff et al., 1995). These results are supported by findings that methylation and repeat length vary by tissue (Wöhrle et al., 1992) and that the CGG repeat length increases in tissues with less methylated repeats in *FMR1* (Field et al., 2019). Moreover, repeats of CGG can also expand among generations (Cronister et al., 2008), which might be a result of demethylation in the early embryonic stage.

Interruptions inside the repeats can also influence insertion or contraction. The CAG repeats of patients with amyotrophic lateral sclerosis with more CAA interruptions are markedly shorter than those of patients with fewer interruptions (Yu et al., 2011), which indicates that interruptions are critical for preserving repeat length and preventing further insertion or contraction. The transmission of CGG repeats of intermediate (~46–60 CGG repeats) alleles in the *FMR1* gene is much more stable than that of the more than 200 repeats in FXS (Nolin et al., 2003), which might be because shortening CGG repeats magnifies the interruption effect of AGG codons within the repeats. Moreover, the more stable inheritance of CAG repeats of FVB-Tg(YAC128)53Hay/J than B6CBA-Tg(HDexon1)62Gpb/3J described by Jackson Laboratory might also suggest that the nine extraordinary interspersed CAA codons in CAG repeats of YAC128 act as markers.

The dominant theory proposes that repeat expansion might be brought about by the slippage that occurs during DNA replication (Hartenstine et al., 2000). As normal individuals also have repeats and may experience slippage according to the theory, it is not rational that normal individuals with fewer repeats do not exhibit symptoms of HD and increased CAG repeats. Furthermore, the slippage theory cannot explain the positive association between increased CAG repeats and expression of the *IT15* gene. Moreover, the slippage mechanism also lacks direct proof *in vivo* (Liu and Leffak, 2012). Thus, other mechanisms may provide a better explanation. Given the association between DNA extension or contraction and methylation and sequence specificity, homologous recombination (HR) may be involved in genome changes, which could be inhibited by methylation and only occur when two double-strand DNAs (dsDNAs) on the genome are of sufficient similarity. Moreover, HR can be efficiently enhanced by transcription (Gottipati et al., 2008). But how is HR involved?

## The Role of Alu Elements, Pre-mRNA Splicing, and HR in CNV Extension

There are thousands of CNVs distributed on the genomes of normal human individuals (Iafrate et al., 2004; Sebat et al., 2004; Redon et al., 2006; Korbel et al., 2007). Because of their differences among races and populations (Sudmant et al., 2015), CNVs have gained more and more attention in recent years. Amplification of specific genes results in abnormal CNVs in different kinds of tumors (Slamon, 1990; Lovekin et al., 1991; O'Reilly et al., 1991; Paterson et al., 1991; Niu et al., 2020). As tumor cells originate from normal cells, possibly with normal CNVs, the conversion of tumor cells indicates that the genome might change dynamically over the life course. Moreover, as the expression of genes within CNVs tends to correlate with copy number changes, and CNVs vary among tissues (Henrichsen et al., 2009) differentiated from one fertilized egg, changes in CNVs should play a critical role in the determination of differentiation and thus expression patterns in different tissues. It is important to note that dosage alterations of genes expressed in the brain, which are created by corresponding CNVs, are less frequent than those of other genes (Henrichsen et al., 2009), which strongly indicates that changes in CNVs might be associated with cell division, because the neuron ceases to split almost after birth. CNVs may also be altered relative to expression throughout one's life, similar to the insertion of repeats in neurodegenerative diseases.

The transcription of mRNA is directly associated with gene expression. During transcription, many lariats are produced with functions unclear because of the existence of introns. In addition, copies of genes within CNVs are homogenous and retain their original functions (Nei and Rooney, 2005; Romero et al., 2017). Thus, the mechanism involved in extending CNVs should be able to contain almost all of the information of an entire gene. But how transcripts of genes take part in CNV extension still needs to be determined.

Is this possible? Quantities of circular intron RNAs (ciRNAs) are detected through sequencing, and most are small in size, with a length <500 bp (Panda et al., 2017). Although most ciRNAs found in this study are located at the 5′ end of the introns, this might be because ciRNAs that originate far away from the 5′ end of the introns would be discarded due to the alignment in sequencing, as the efficiency of RNA sequencing for identifying circular RNAs is lower than that of the microarray (Li et al., 2019). Moreover, the location of ciRNAs at the 5′ end of the introns suggests a relationship between ciRNAs and lariats processed from introns (Panda et al., 2017). Moreover, most ciRNAs are created from processed lariats discarded from the splicing of pre-mRNA (Zhang et al., 2013). These results together indicate that the lariats might not be as long as previously thought and thus recursive splicing should exist widely. Many splice donor sites embedded within introns demonstrate the existence of recursive splicing (Kelly et al., 2015). Furthermore, alternative splicing occurs on more than 90% of genes on the human genome (Pan et al., 2008; Wang et al., 2008).

In addition, more than half of the exons in human *IT15* and *FMR1*, perhaps the whole genome, have the AG binucleotide on their 3′ end or GT or GC on their 5′ end, whereas others do not, which indicates that the GT, GC, or AG binucleotide on the flanks of exons might not be necessary for splicing. As most introns start with GT or GC and end with AG, which are signs of splicing, their frequent appearance on the flanks of exons might indicate that the exons might also be spliced, similar to the introns. Thus, lariats could be generated that overlap with others nearby. Note that the generation of exon-containing lariats might not be just for alternative splicing given the large proportion of exons with the GT, GC, or AG binucleotide on their flanks.

For example, in exons with AG on their 3′ end, the GT or GC binucleotide, a sign of the 5′ end of lariats, does not often appear on the 5′ end. Therefore, lariats with those exons might have their 5′ end fell in the upstream introns with part of the introns spliced. Moreover, in ordinary splicing, exons are spliced completely, and lariats containing only part of the introns upstream can be produced. These lariats would overlap partially with those containing both the exon and upstream intron. The partially overlapping lariats could transfer information from pre-mRNA one after another. Moreover, if lariats containing exons can also be spliced, there must exist a mechanism by which the splicing that generates exon-containing lariats is much weaker than other ordinary splicing events. Thus, a possible mechanism of splicing is proposed.

**Figure 1:**
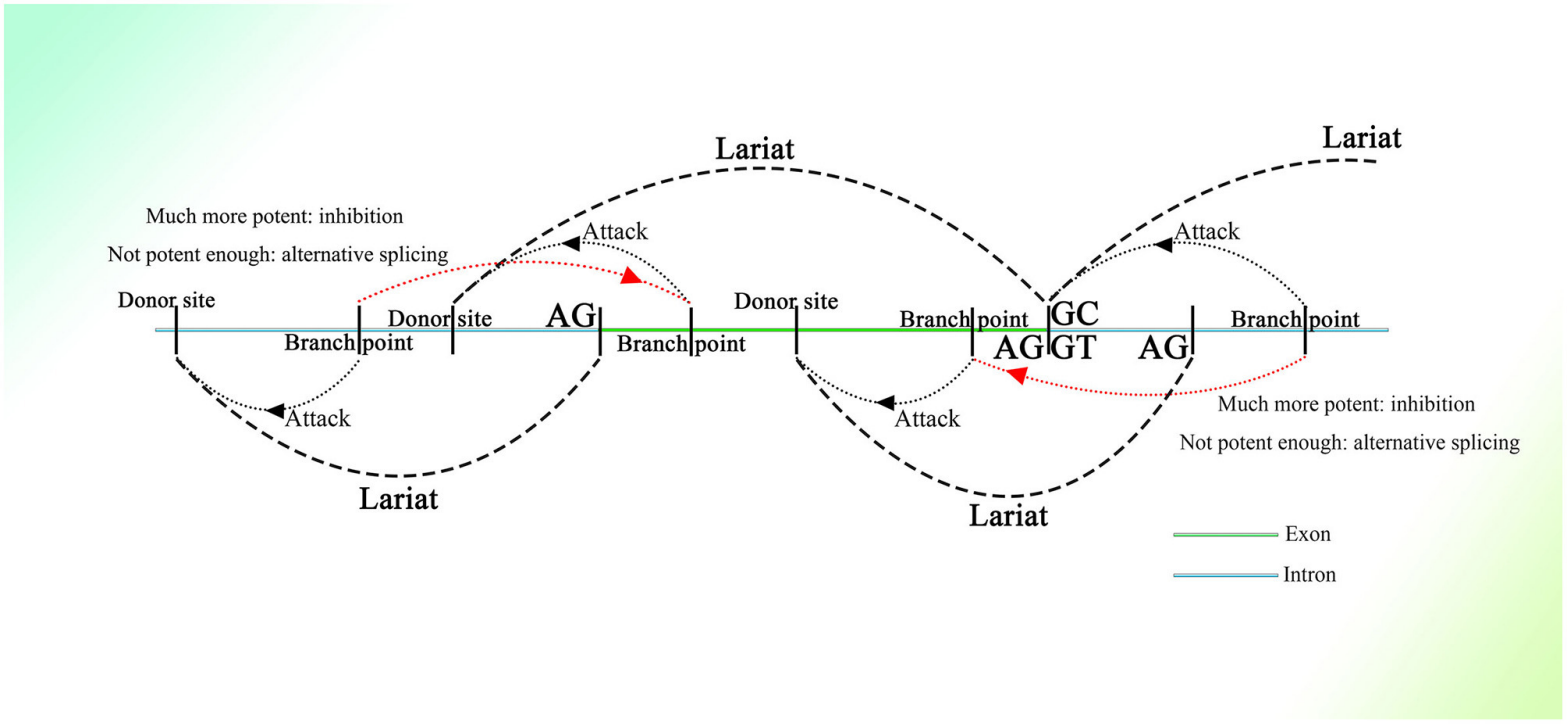
Schematic diagram of the generation of partially overlapping lariats. The branch points and donor sites are distributed on the genome. The branch points close to the exon are much more potent than those closest to or on the exon ordinarily, which leads to the excision of the exon, whereas if they are not potent enough, the exon will be excised as alternative splicing. Because the exon-containing lariat also has part of an intron within it, mRNA with the exon sequence only is not able to be spliced because of the loss of the intron.

Many donor sites might be located within introns and exons with short intervals. At the same time, branch point sites lie between two donor sites, and all of them are activated by different strengths of splicing initiation. The branch point could jump across a nearby acceptor site on an upstream sequence and attack the donor site further. Thus, partially overlapping lariats could be produced all over the transcriptional region. As for the excision of exons, the branch point taking charge of the splicing on the 3′ end of the exon would be much more potent than the branch point upstream closer to the 3′ end, of which the donor site might be located on the upstream sequence of the 3′ end. Thus, the potent branch point could lead to successful splicing at the 3′ end of the exon, suppressing adjacent competitors that would splice the exon, similar to the “overdrive suppression” mechanism in cardiac conduction (Figure 1).

Similar things would happen on the 5′ end of exons, as the binucleotides on the 5′ end are not always GT (their splicing strength should be weak even if they are GT), and they would not be seen as donor sites, and the entire exon could be excised successfully with high probability. But splicing mediated by branch points closest to the 5′ end of exons could still happen with small probability, producing exon-containing lariats that overlap with the ordinary ones without exons described above (Figure 1). In addition, inhibition of the formation of splicing intermediates could influence efficient exon–exon splicing (Kelly et al., 2015), which also indicates the existence of competition between one branch point and its corresponding neighbor branch point and the presence of exon-containing splicing intermediates, although few in number.

In this splicing mechanism, all branch points would compete with other neighbor branch points, resulting in different series of splicing. But most of the time, the series of splicing activity would be initiated by the most potent branch points, which are always those responsible for the exon excision; thus, the other series of splicing partially overlapped by them would be suppressed and would occur at a much lower frequency (Figure 1). The wide distribution of AGGT or AGGC with short intervals on the genome and the variety of branch point sequences with different possibilities of being recognized (Harris and Senapathy, 1990) provide evidence of this mechanism. Moreover, this is supported to a certain degree by evidence that the intron could also be processed from its 3′ end (Pandya-Jones and Black, 2009).

Other results might also indicate the existence of such a splicing mechanism. Longer introns markedly increase the time required for transcription (Swinburne et al., 2008), which indicates that the process takes time with longer introns and that the introns are not cut entirely at once. Moreover, the fact that exon skipping happens before the transcript is released into the nucleoplasm (Pandya-Jones and Black, 2009) also suggests that all parts of pre-mRNA are processed under the same rules at the same time, including the overdrive suppression of splicing that determines the retention of exons. The mechanism is also supported by the fact that in some introns recursive splicing is the predominant method of splicing, even in shortened introns (Burnette et al., 2005).

This mechanism might be difficult to identify, as splicing intermediates are hard to detect because of their short half-lives (Burnette et al., 2005; Kelly et al., 2015), and fewer than 100 lariats are characterized *in vivo* (Taggart et al., 2012). The main approaches used to identify splicing intermediates are sequencing and polymerase chain reaction (Burnette et al., 2005; Taggart et al., 2012; Kelly et al., 2015). Sequencing results are aligned with the exon–intron junctions annotated in the genome, which might largely ignore the lariats produced far away from the junction. Moreover, given the existence of processes from the 3′ end of introns (Pandya-Jones and Black, 2009), sequencing aligned with the 3′ end of exons and polymerase chain reaction that uses the 3′ flank of exons as primers can hardly detect splicing activity.

Although this splicing mechanism provides enough information to extend the transcriptional parts of genes, the recombination still needs the structure of dsDNA, and only reverse transcription can convert the single-strand RNA (ssRNA) into dsDNA.

**Figure 2:**
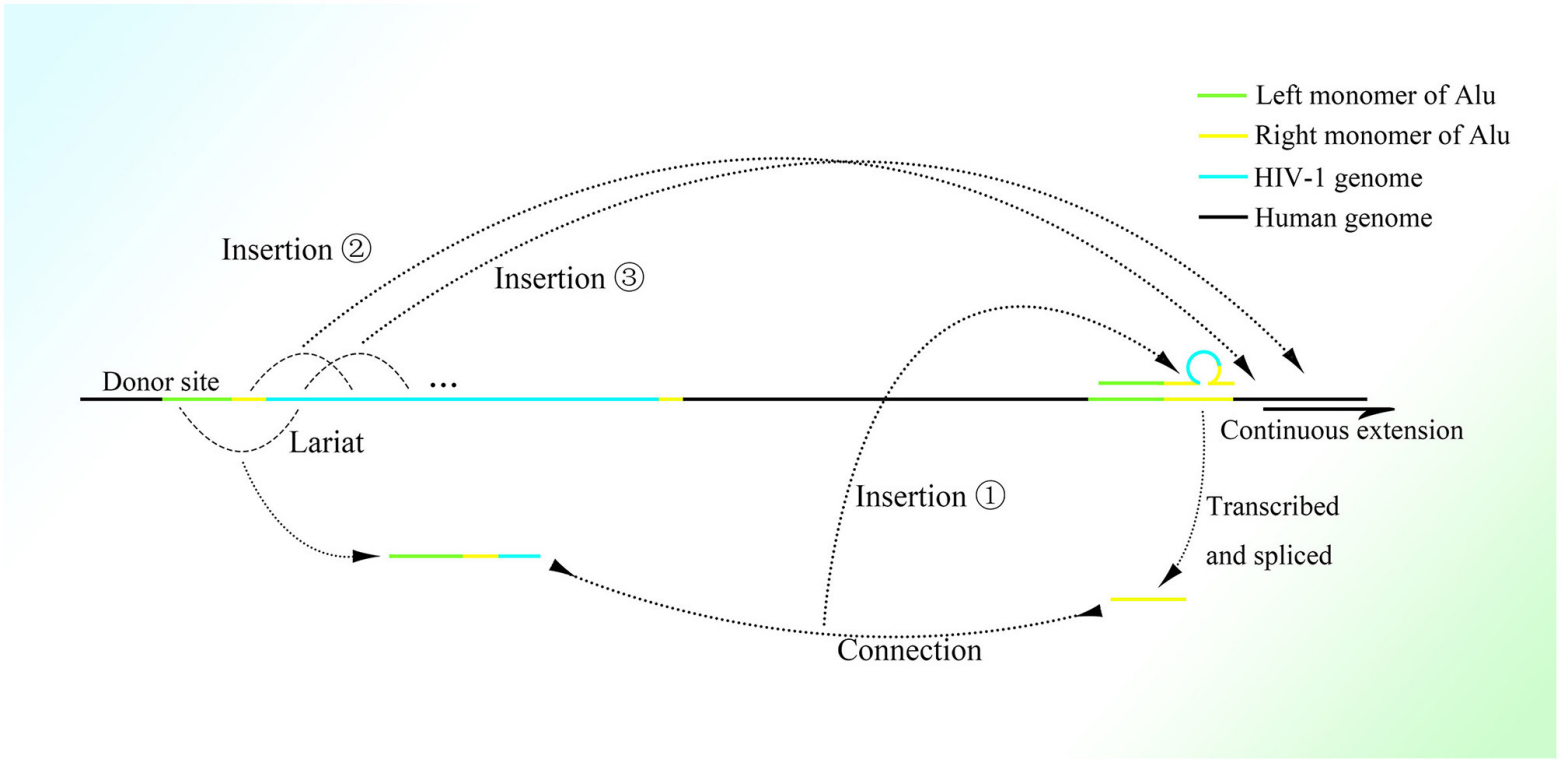
The possible mechanism of insertion of the HIV-1 genome into the right monomer of the Alu element. A lariat containing the left monomer of the Alu element, part of the right monomer, and the 5′ sequence of the HIV-1 genome would be produced and connected to the right monomer of Alu transcribed and spliced nearby. Then the 5′ region of the HIV-1 genome would be inserted into the right part of another Alu element nearby with the assistance of the lariat-Alu complex. The remaining HIV-1 genome downstream would be gradually inserted at the new insertion site within another Alu element.

Any mRNA transcribed in feline cells can be reverse-transcribed and retrotransposed with the help of open reading frame 1 protein (ORF1p) and open reading frame 2 protein (ORF2p), which strongly indicates the participation of retrotransposons in transcription-related genome change (Esnault et al., 2000; Dewannieux et al., 2003). Moreover, the presence of a polyadenine (polyA) stretch on the 3′ end of the retrotransposed mRNA sequence (Maestre et al., 1995; Esnault et al., 2000) suggests the participation of Alu elements, which also have a long polyA tail on the 3′ end. The Alu sequence might not be detected in these insertions because HR between the mRNA polyA tail and the Alu 3′ A-stretch, which could be of high efficiency with the pure adenine sequence, deletes the sequence of the Alu right monomer between them. It is also possible that retrotransposition is mediated by ORF2p combining with the polyA stretch of mRNA (Dewannieux et al., 2003). Moreover, the preference of the genetic material of human immunodeficiency virus (HIV) to be inserted into Alu elements of genes with higher transcriptional levels (Cohn et al., 2015) might relate the functions of Alu elements to expression (the detailed mechanism is depicted in Figure 2). Moreover, the same combination of reverse transcription and lariats as in the group II intron has been observed in many simple organisms (Guo et al., 1997, 2000; Singh and Lambowitz, 2001; Jiménez-Zurdo et al., 2003). Alu elements are more clustered in genes involved in metabolism, transport, and signaling processes but much fewer in number in genes related to information pathway component coding as well as structural proteins (Grover et al., 2003), which strongly indicates that Alu elements are associated with cell functions. Alu elements are one kind of short interspersed element and retrotransposon that constitutes a large proportion of the genome, with more than 1 million copies (Lander et al., 2001); only one fourth of genes do not have Alu elements (Grover et al., 2004). Moreover, Alu elements are widely distributed on the genome, with about a 4,000 bp interval on average (Smit, 1999). They consist of two similar but different monomers linked by an A-stretch, with a much longer A-stretch at the 3′ end, and only the left monomer has active promoter to start transcription by RNA polymerase III (Quentin, 1992a,b). In addition, many CpG sites distributed on Alu elements result in much higher methylation than other areas on the genome (Greally, 2002; Khitrinskaia et al., 2003). The retrotransposition of Alu elements can be initiated by recognizing the sequence TTAAAA and then splicing one strand of dsDNA on the genome with the help of the ORF2p transcribed by LINE-1 (Mathias et al., 1991; Wallace et al., 2008), converting the Alu element from ssRNA into ssDNA with the spliced single DNA strand as primer. This process is called target primed reverse transcription. But the next step in the synthesis of the second strand of dsDNA and its integration into the genome still remain unclear.

Because both recombination and reverse transcription mediated by Alu elements can only nick one of the dsDNA strands, some relationships might exist between them. The dsDNA generated by transposons could be integrated into the genome with the aid of a mechanism closely related to HR, as expression of LINE-1 would create γ-H2AX foci, which could also be seen in recombination (Gasior et al., 2006). Furthermore, motifs similar to translin-binding sites are also identified within the Alu element consensus (Jeffs et al., 1998). Because translin is a recombination hotspot binding protein that specifically binds to consensus sequences at the breakpoint junctions of chromosomal translocations (Aoki et al., 1995), the functions of Alu elements might be related to recombination. Moreover, the presence of a common 26 bp core sequence in Alu elements at or close to recombination sites and the sequence within it similar to the 8 bp sequence known to mediate recombination in *Escherichia coli* (Rüdiger et al., 1995) might also suggest the involvement of recombination in the functions of Alu elements.

Some critical characteristics of Alu elements might provide important clues. The secondary structure of the right monomer of an Alu element transcript constitutes a structure similar to Ω, retaining a gap between the two complementary sequences. The conserved sequences of UU and AAAA are located separately on the two feet of Ω just close to the gap (Häsler and Strub, 2006). This strongly indicates that ORF2p might exert its endonuclease function with the combination of these sequences and that the Ω structure that the sequence recognizes by it on the genome is just TTAAAA. The exertion of endonuclease by ORF2p would demand the sequence TT, AAAA, and the gap between them with the Ω-like structure, which indicates that splicing on the single strand of dsDNA could only happen when there is no direct connection between the two sequences used for recognition and implies that the extraordinary sequence on the circle of Ω waits for insertion. The reason why ORF2p on the Alu transcript recognizes TTAAAA most of the time might be that something, such as the Ω structure blocks the advance of ORF2p from 3′ to 5′ (the direction of reverse transcription), with its specific structure making ORF2p stay with UU and AAAA on the Alu transcript. Furthermore, ORF2p on LINE-1 could also recognize and nick other sequences besides TTAAAA (Cost and Boeke, 1998) providing it the ability to nick elsewhere. Given the recognition characteristic of ORF2p on the dsDNA sequence complementary to that of nucleic acid it combines with (Cost and Boeke, 1998), it is reasonable to think that when ORF2p moves to the 3′ end of ssDNA synthesized by itself with reverse transcription, it could without impediment move from 3′ to 5′ freely with the recognition of complementary sequences, searching for six nucleotides on Alu ssDNA with a gap complementary to the genome and then splicing the site on the genome corresponding to the gap on the Alu product. Note that the special secondary structure Ω of Alu elements cannot be seen in other nucleic acid guidance of endonuclease, such as clustered regularly interspaced short palindromic repeats, which suggests that it might be of special significance.

Moreover, the half-lives of Alu element transcripts are quite short, as they can be spliced at fixed sites before the middle A-stretch, leaving a product called small cytoplasmic Alu, which contains only the left monomer of the Alu element without the middle A-stretch and remains much longer than the full-length transcript (Matera et al., 1990; Maraia et al., 1993; Chu et al., 1995). It seems that the part that disappears receives less focus because of its short half-life.

**Figure 3:**
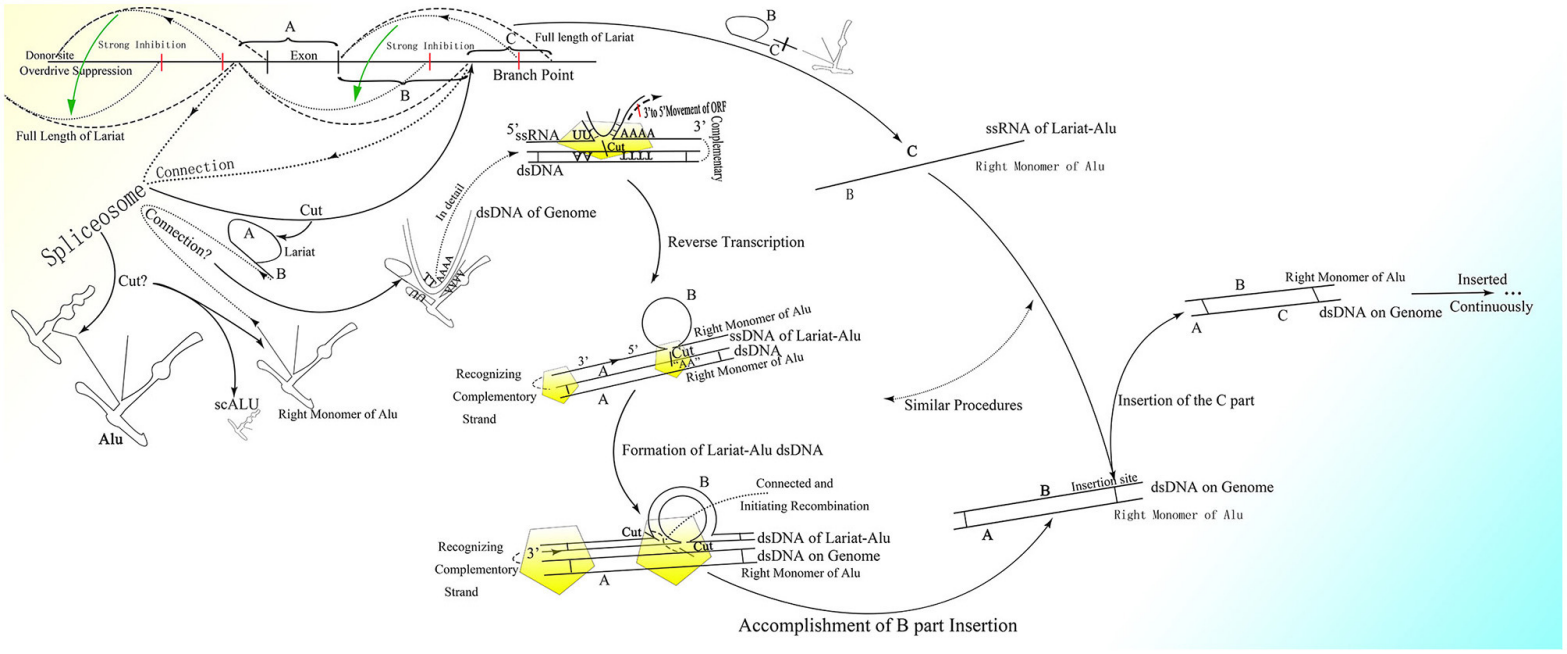
The detailed process of the CNV extension mechanism. Partially overlapping lariats would be produced during pre-mRNA processing with exons excised of high probability by overdrive suppression. With the pre-mRNA spliced and connected, the right monomer of the Alu element could be spliced and connected to the lariats produced at the same time. With the help of ORF2p on the right monomer of the Alu element, the lariat-Alu ssRNA could be converted into dsDNA at the specific insertion site, with part A on the left and the right monomer of Alu on the right. Then part B would be inserted into the genome facilitated by HR with the two nicks produced by ORF2p. Part C would be inserted further by lariats containing parts B and C at the specific insertion site between part B and the right monomer of the Alu element. CNVs would be extended continuously and spontaneously through this mechanism.

It is reasonable to deduce that the part containing the middle A-stretch and right monomer could be connected to the lariats spliced from pre-mRNA forming lariat-Alu ssRNA. As the 5′ end of lariat is concealed, the right monomer could only be linked to the 3′ end of the lariat through its 5′ middle A-stretch. With the help of the right monomer of the Alu element and the endonuclease ORF2p on it, the lariat fragment could be reverse-transcribed to single-strand DNA (ssDNA) with the part carrying information from the genome on the 3′ sequence, with the right monomer of the Alu element on the 5′ sequence. When there is a specific site on the genome with part of the lariat sequence at its 5′ side directly connected to the sequence of the middle A-stretch and the following right monomer at the 3′ side, the lariat-Alu ssDNA could combine with it and form a Ω structure with a gap between the two feet because the middle part of the lariat-Alu ssDNA is lacking at the site on the genome. With the help of ORF2p recognizing the complementary sequence on the genome from the 3′ end of the lariat-Alu ssDNA containing genome information, one of the strands on the genome would be spliced at the site with the lariat-Alu ssDNA complementary to the genome on the two sides of the site, and thus the lariat-Alu ssDNA would be converted into dsDNA through a process similar to reverse transcription. Note that only when the two sequences are fully complementary can ORF2p move from 3′ to 5′; this is similar to DNA polymerase, in that further DNA synthesis requires a synthesized sequence complementary to the downstream sequence. Then the lariat-Alu dsDNA would be preserved until the cell starts to split, DNA replication is finished, and HR is permitted. During this phase, ORF2p could still recognize the same site and then splice the genome again, producing a nick on the same strand spliced before. At the same time, because the lariat-Alu is converted from ssDNA into dsDNA, ORF2p could splice the other strand of lariat-Alu dsDNA, which does not exist in the status of ssDNA before. The two nicks produced would facilitate the occurrence of recombination, mediating the insertion of the sequence on the circle of Ω into the genome (Figure 3).

In addition, the synthesis of the second DNA strand and dsDNA integration might not happen at the same site as reverse transcription, which could make the retrotransposition mechanism confusing. This process might be similar to reverse transcription of HIV, which happens at different sites in different steps as strand transfer (Zheng et al., 2005). Because the left monomer of the Alu transcript is lost during the formation of lariat-Alu ssRNA, the insertion would not be able to occur at the 5′ end of the integral Alu element within genes on the genome, which might result in incorrect insertion.

Although the lariat-Alu dsDNA would not be able to initiate HR with the genome with high efficiency because it is exogenous compared to the genome and might not be able to recruit proteins, such as Rad51 to mediate HR, ORF2p could substitute these proteins to a degree, creating two nicks, which is necessary for HR. But because ORF2p is unable to completely mediate integration, part of the system used in HR might still be required, and thus insertion could only happen during cell division after DNA replication. Thus, it could be deduced that every split of the cell may bring about genome change, although slight.

Therefore, with guidance from overlapping lariats, CNVs could be extended continuously and gradually with the information carried by pre-mRNA (Figure 3). Note that only when the upstream fragment is inserted and the new insertion site appears would the insertion of the fragment downstream be able to occur. Thus, every cell division might bring about specific genome change according to transcription. Given the shortness of lariats, the genome could only be inserted with short sequences and changed slightly, which would be hard to detect during cell division. The new cells divided from the old one would be relatively and slightly different from their parent.

From the CNV extension mechanism, it could be inferred that there should be a right monomer of the Alu element at the end of CNV extension on the genome (Figure 3). The existence of the free right Alu monomer on the genome (Quentin, 1992a,b) provides evidence supporting this deduction. Moreover, the ends of CNV extension differ in different cells and change with cell division, which is why they are difficult to detect (Jurka and Zuckerkandl, 1991).

Only genes with sufficient expression could be expanded, as the splicing of uncommon lariats that overlap partially with ordinary lariats is much weaker than that of ordinary ones and requires sufficient quantities of mRNA. Hence, information on gene expression could be fed back to the genome to help determine the changes to the CNVs on the genome. The comparably short but sufficient length of the lariats provides sufficient sequence difference among the lariats, which could greatly diminish non-specific binding and thus make DNA insertion efficient and specific. Besides gene expression, the transcriptional level of Alu elements within corresponding genes might also influence the efficiency of insertion, in that although multiple active Alu element loci could be detected, the transcriptional level of Alu elements would differ among different kinds (Shaikh et al., 1997).

Finally, the unfinished pre-mRNA transcribed from the incomplete CNVs could be transferred to the entire gene to complete transcription of the remaining part. Thus, although the extending CNVs could not be transcribed entirely before the full copy extension is accomplished, the corresponding gene expression would still increase with the extension in CNVs. As the 5′ flanking region could not be transcribed, insertion of this part might be mediated by 3′ transduction effects of LINE-1 elements (Matsutani, 2006), which are described in detail below.

Similar splicing of the retrotransposon could also be observed in mice. B1 in mice, which corresponds to Alu elements in primates, can be spliced into two parts as well, with one of the products possessing a promoter and the other having an A-stretch on the tail (Maraia, 1991). The splicing, by which the 5′ sequence containing the promoter as small cytoplasmic Alu is cut off from the integral Alu transcript, leaving the remainder to connect with lariats, might aim to prevent the retrotransposon from being transcribed at the insertion site, which could link to the lariats produced nearby without downstream information and obscure the correct insertion. Furthermore, deletion of the 5′ sequence of retrotransposons also avoids the wrong insertion at the 5′ end of integral retrotransposons on the genome, and thus the insertion could only occur before the specific right monomer of the Alu element as the end of CNV extension, extending the CNV.

Moreover, the instability of CAG repeats in HD is correlated with the cell cycle pathway but not the expression of DNA repair genes (Lee et al., 2010), which also fits the association between genome change and HR. The transformation of gene expression and status in cultured cells after passage might be brought about by this mechanism as well. As the situation *in vitro* would affect the gene expression style, the genome of the cultured cells would gradually become different from those *in vivo* with different CNVs extension. Moreover, the expression-correlated trinucleotide expansion in neurodegenerative diseases described above could be seen as gene extension according to transcription from another aspect, which could be an example of the CNV extension mechanism.

In addition, as spliceosomes can splice and link the ends created during pre-mRNA processing with their comparably complex structures (Wan et al., 2019), they might also be able to splice Alu elements and link them to lariats through similar procedures. Meanwhile, the combination of two functions on one protein as ORF2p (Hohjoh and Singer, 1996; Kulpa and Moran, 2006) indicates that splicing effects could be exerted elsewhere with the movement brought about by reverse transcription. Because ORF2p is recruited at a fixed site as Ω, its following forward direction and splicing effects would be under control. Furthermore, ORF2p could only combine at certain site on the Alu transcript and exert specific and different functions with movement by a single protein. Thus, even though ORF2p exists at low levels in nuclei, this insertion mechanism could still work effectively. Meanwhile, the low expression of ORF2p would avoid its non-specific combination as well, as ORF2p might also combine with the polyA stretch of mRNA and mediate retrotransposition at a much lower rate than that of Alu (Dewannieux et al., 2003).

**Figure 4:**
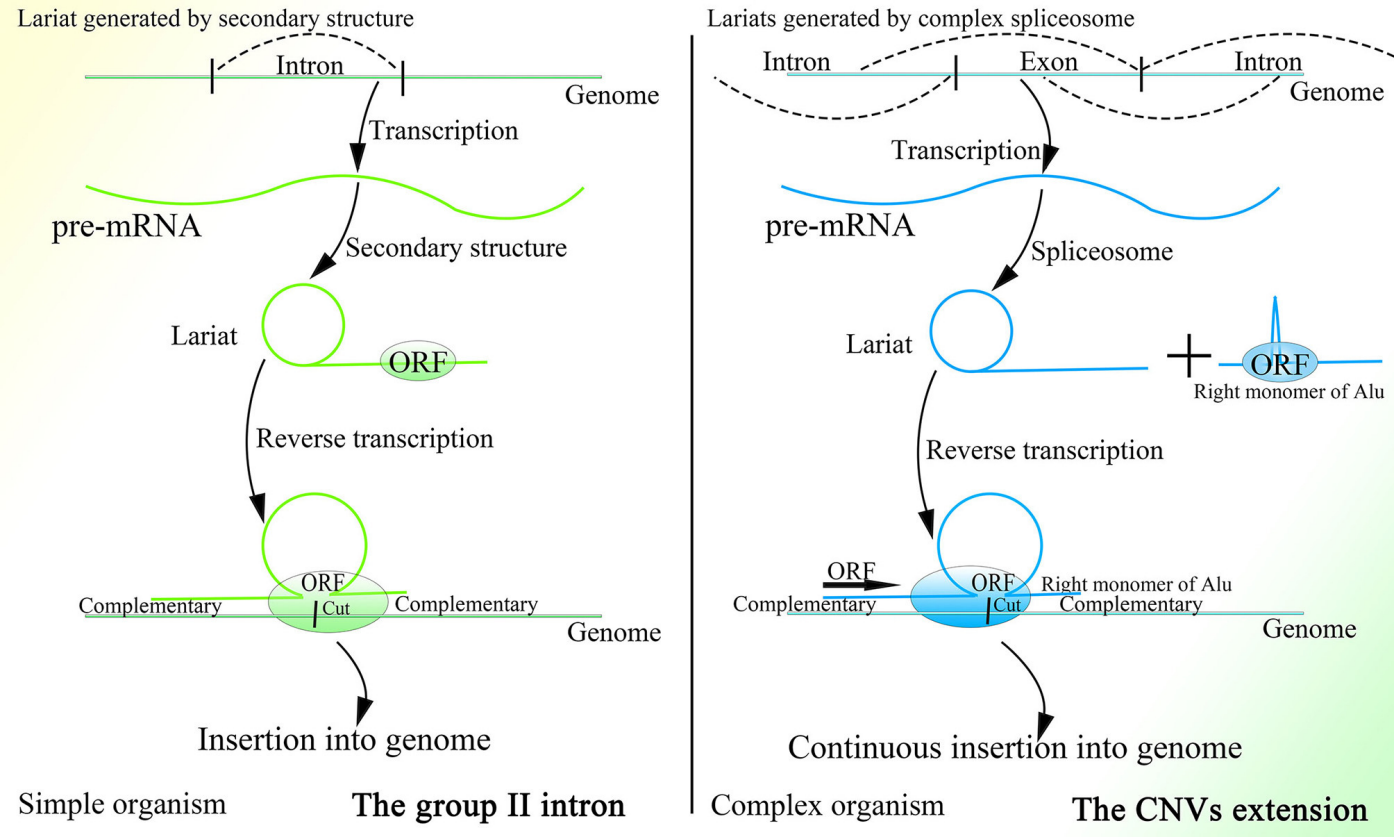
A comparison of the group II intron and the CNV extension mechanism. Given the existence of spliceosomes in complex organisms, the various lariats would be produced without the help of the specific secondary structure, and thus CNV extension brought about by continuous insertion would be possible.

It is interesting that this mechanism is similar to that of the group II intron to a degree, as both mechanisms are based on the retrotransposon and lariat, produce the Ω structure with the two feet complementary to the genome, depend on protein with functions of endonuclease and reverse transcription to initiate insertion, and recognize more detailedly on the 5′ sequence than the 3′ sequence of the genome (Figure 4). In addition, the less conservative sequence of the group II intron might also enable it to mediate continuous insertion on the genome (Guo et al., 1997, 2000; Singh and Lambowitz, 2001; Jiménez-Zurdo et al., 2003). The secondary structure of the group II intron might no longer be needed in higher organisms, as the complex spliceosome (Wan et al., 2019) would substitute it to recognize splice sites and branch point sites. Because ORF2p cannot splice dsDNA and mediate integration, the insertion would be mediated by HR without dsDNA breakage, which makes this mechanism safe for wide application in higher animals. Whether the group II intron is a former version of this CNV extension mechanism requires further research. Surprisingly, the transcription-associated recombination is ubiquitous from prokaryotes to higher eukaryotes and involves gene conversion (Gottipati et al., 2008).

It is rational that the existence of this CNV extension mechanism might explain why HR could occur only during the S or G2 phase of cell splitting and be retained after birth. If HR occurs only to repair DNA damage, then it should be permitted in every phase of the cell cycle to increase cell viability. It was reported that the transcription-associated recombination could only enhance recombination in cells that are in the S phase of the cell cycle (Gottipati et al., 2008). Depending on the CNV extension mechanism, information from gene expression could be preserved in the lariat-Alu dsDNA and ultimately be fed back to the genome during cell division. Moreover, limiting CNV extension by cell division could also make changes in the genome and cell status, which might result in embryonic development and senescence after birth, controllable.

Moreover, each kind of Alu element is prominently clustered in certain areas of the human chromosome, which are relatively fixed among individuals (Filatov et al., 1987), which implies that the effects of elements are regionally specific. Thus, the regional specificity of Alu elements as well as the low transcriptional level (Sinnett et al., 1992; Liu et al., 1994) could largely increase the specificity of their effects in certain areas of the genome and limit their functions within a comparably small range.

As CNVs can be extended, they conversely should also be able to contract on the genome. The same kind of Alu elements clustered together makes possible the deletion of fragments between two Alu elements of the same kind by HR. A report demonstrated the possibility of using HR to delete the sequence between two homologous sequences (Ouyang et al., 2007). If methylation, the main impediment of recombination (Cartwright et al., 1998; Gupta et al., 1998), is removed, HR between two Alu elements distributed closely with similar sequence on the genome might be highly efficient. But the abundance of CpG sites within Alu elements leads to hypermethylation (Khitrinskaia et al., 2003; Saeliw et al., 2018) and thus largely prevents the occurrence of HR among Alu elements after birth and throughout one's life, leading to the irreversible cell status. Deletion can ordinarily only happen during the early embryonic period, in which Alu elements are largely demethylated (Guo et al., 2014; Smith et al., 2014) and CNVs are lost with demethylation (Zhu et al., 2018), and is greatly prevented with the irreversible methylation of Alu elements thereafter. But HR among Alu elements might still occur in the elderly with decreased methylation (Doshi et al., 2004; Bollati et al., 2009), which could lead to oncogenesis. Meanwhile, the consensus sequence within Alu elements might also facilitate the occurrence of HR (Aoki et al., 1995; Rüdiger et al., 1995; Jeffs et al., 1998).

It is rational to deduce that there might exist original copies of genes that could not be altered in any situation, and thus CNVs could be extended according to these original copies. In this way, the genes could be divided into original copies and corresponding CNVs, which are duplicates of the original copies and could be inserted or deleted to change the cell status. As the initial genome sequencing did not detect clear CNV end at which part of the gene is followed by the incomplete Alu sequence, we infer that the duplicates are located upstream to the original copies and thus the sequencing might ignore the CNV end between the two same sequences. This arrangement would make the CNV end always close to the original copies, which may be convenient for the original copies to check the newly inserted fragments. Moreover, the dynamic change of CNV end would also make the detection difficult. The existence of DNA methyltransferase and *de novo* methyltransferase but not only one kind in most eukaryote (Bewick et al., 2017, 2019) supports the possibility of DNA insertion and deletion with the preservation of the original copies of genes, as the loss of *de novo* methyltransferase can still sustain the existence of species (Catania et al., 2020). As the extending CNV is not integral, the RNA transcripts transcribed by it would be transferred to the original copies or integral CNV to finish transcription. A series of proteins, such as Dcp1a and Xrn2, which can be recruited to certain sites on the genome, are able to pause transcription (Brannan et al., 2012) and interact with TTF2 (Leonard et al., 2003; Brannan et al., 2012), a kind of protein that can release pol II and RNA transcripts from the DNA template (Liu et al., 1998). Thus, it is reasonable to deduce that at the end of the extending CNV, just after the right part of Alu, there might exist similar sites that could recruit Dcp1a, Xrn2, and some other proteins. As the CNV extends, the transcriptional level of the corresponding gene would gradually increase.

As for genes without an Alu element, such as *GAPDH*, because they are not able to contract and conversely be extended, it is not necessary for their introns to be enlarged to regulate the change rate of corresponding genes, which ultimately results in the comparably short introns within these genes and their steady expression under any conditions. The introns within these unchangeable genes might aim to separate potential splice sites and branch points on coding sequence (CDS), to prevent CDS from being spliced by the spliceosome. The splicing strength of introns should be much more potent than that of exons and CDS. Therefore, these introns could act as decoy missiles; The spliceosome would attack the introns first, and the derived CDS would be spared. Prokaryotes can directly transcribe CDS without introns, possibly because of their lack of spliceosomes. The existence of spliceosomes might force every gene to contain introns, and conversely the fact that almost all genes in higher animals contain introns indicates that exons and CDS could be spliced as well. Moreover, the splicing mechanism also brings about various variants of mRNA.

CNVs continue to be sought out, and more and more CNVs are being detected. At the same time, detecting CNVs is still tough. According to the proposed CNV extension mechanism, there should be an end of the extending CNV, at which part of the gene is followed by the right monomer of the Alu element. If the search for CNVs focuses on this specific area, it might become easier. We recommend that these dynamic sites be aligned in sequencing and thus detailed CNVs extension conditions be further studied. Moreover, because the fragments inserted in CNVs extension are small in size, the CNV extension ends could differ among different cells. Sequencing might not be accurate enough to track every insertion event. We may detect the lariats' sequence through the labeled Alu transcript; the ends of CNVs extension are different combinations of part of various genes and the right monomer of Alu. Depending on the known ends, the microarray and PCR could be more sensitive and effective.

In addition, given the short half-lives of lariats and low expression (Liu et al., 1994; Doshi et al., 2004) of Alu elements, together with their widespread existence and dispersed distribution throughout the whole genome, the connection between the right monomer of Alu elements and different lariats would be hard to detect and discriminate from ordinary pre-mRNA. Although we can detect the splicing of lariats to a degree, the expression of Alu elements is far less than that of lariats, which results in only a very small number of lariats being connected. Only when the Alu transcript is spliced and its 5′ end is exposed could it be connected to a lariat, and meanwhile the 3′ end of the lariat would be degraded in a short time, converting the lariat into circular RNA (Zhang et al., 2013) or degraded totally, making this process accurate and hard to track. Moreover, the transcription of LINE-1 transposons, which is necessary for the transposition of Alu elements, can be detected from normal somatic cells (Belancio et al., 2010), which suggests that Alu elements are required for proper functioning. By the way, this CNV extension mechanism can provide rational explanations for several unsolved questions in evolution, embryonic development, senescence, and oncogenesis, which are described in detail below.

## Possible Trace of Expression-Related dsDNA in Normal Individuals

Late in the last century, a kind of DNA fragment was found in sperm and named sperm DNA fragmentation (SDF) (Evenson et al., 1980). Research ultimately showed that an increase or decrease in SDF can indicate decreased fertility (Spanò et al., 2000; Loft et al., 2003; Evgeni et al., 2015). Sperm with normal functioning still exhibit a degree of SDF, even though it is unbelievable that the genome of a haploid cell could be passed down entirely with parts lost and converted into SDF. Moreover, increased SDF is observed in sperm under stress (Muratori et al., 2015). This is in line with the finding that expression of Alu elements can also increase when cells are stressed (Liu et al., 1995). Thus, it is possible that SDF is one of the products of the effects of Alu elements and that increased SDF might be due to a loss of control in sperm with more insertion sites and insertion events. Because both insertion and deletion depend largely on methylation, the increase in insertion sites might indicate disorder, which is consistent with the fact that senescence can lead to disordered methylation (Bollati et al., 2009) and promote the production of SDF (Tirado et al., 2009). In addition, the visible SDF of sperm might originate from the increased lariat-Alu ssDNAs or dsDNAs brought about by the broader and higher transcriptional level of sperm than other cells (Soumillon et al., 2013; Xia et al., 2020).

Besides, it was reported that a kind of extrachromosomal circles named microDNAs, which are <400-base in length, are abundant in all tissues of normal individuals (Shibata et al., 2012; Dillon et al., 2015). The length of microDNA coincides with that of lariat-Alu dsDNA and the cyclization of microDNAs would cause partial deletion. Moreover, the generation of microDNAs is also linked to transcriptional activity and splicing (Dillon et al., 2015), which is consistent with the characteristics of lariat-Alu dsDNA production. The fact that microDNAs tend to overlap more with exons than with introns (Dillon et al., 2015) might be caused by the different splicing strength between the regions upstream and downstream to exons, which could lead to the alternative splicing. Furthermore, a large percentage of microDNAs contain short interspersed nuclear elements, such as Alu elements and CpG islands, which are also enriched in Alu elements (Dillon et al., 2015). In addition, the fact that the depletion of the SR protein splicing factor ASF/SF2 induces genomic instability and high-molecular-weight DNA fragmentation (Li and Manley, 2005) suggests the interaction between lariat-Alu dsDNA and genome as well. As the splicing factor is inactivated, longer lariats would be produced and finally be converted to high-molecular-weight DNA fragments. Some researches may attribute these phenomena to the R loop structure, but the R loop theory is unable to explain the increased number of specific gene copies with function during embryogenesis and oncogenesis (Henrichsen et al., 2009; Meinhardt et al., 2015; Hannibal and Baker, 2016; Fischer et al., 2017; Shao et al., 2019). Moreover, the relationship between R loop and genomic instability is deduced from the improvement of genomic stability after the degradation of RNA:DNA hybrid, but at the same time, the RNA:DNA hybrid could also appear during the production of lariat-Alu dsDNA, and the elimination of RNA:DNA hybrid would decrease the lariat-Alu dsDNA production and stabilize genome as well.

## The Possible Effects of Dynamically Changing CNVs in Evolution

**Figure 5:**
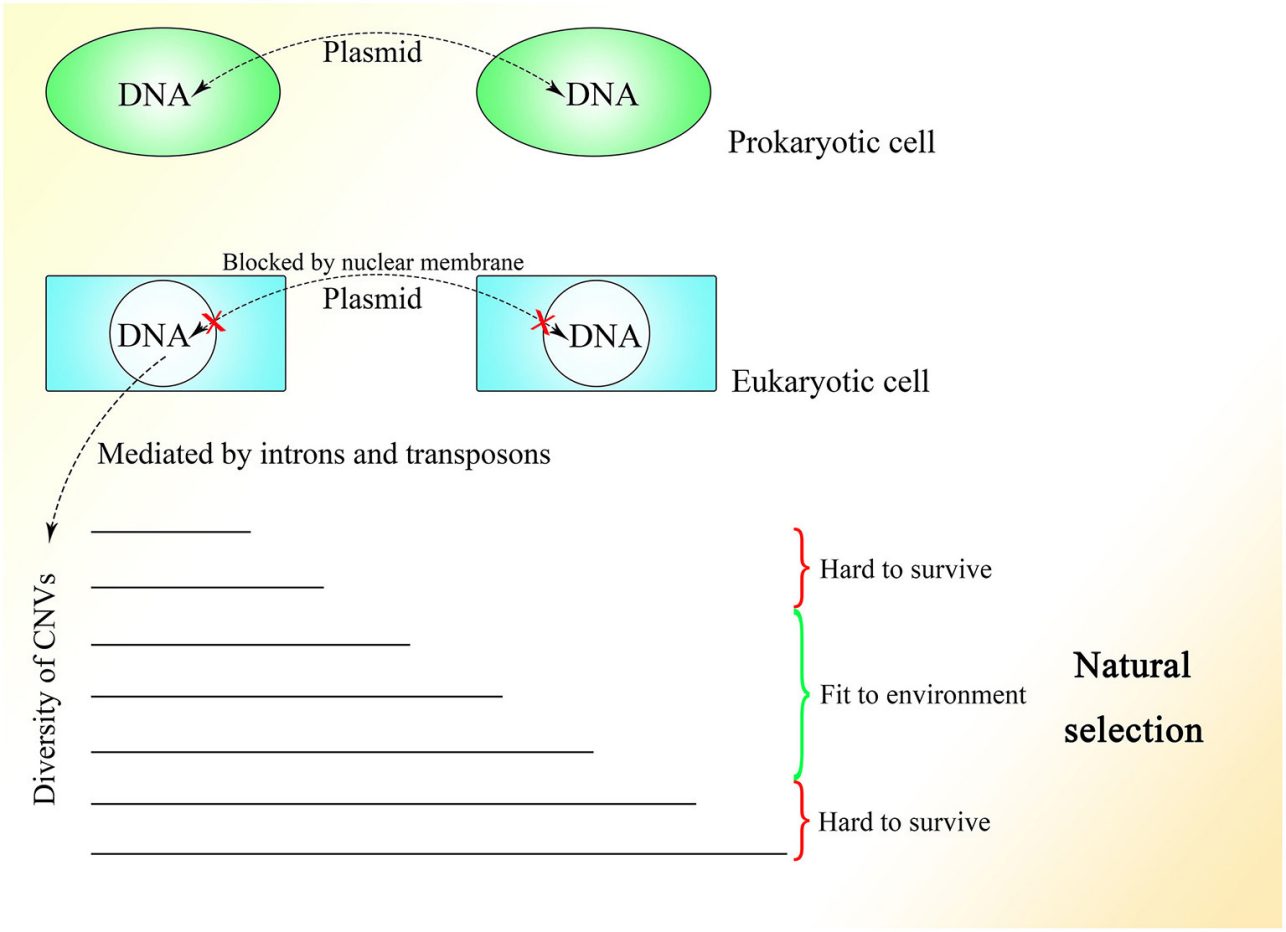
The CNV extension mechanism in evolution. The nuclear membrane of eukaryotes reduces interference from outside the nucleus, creating the environment required for stable and precise CNV extension under control while introns provide various CNVs with different lengths under regulation. The CNVs of different individuals in the same group could be oscillatory, and natural selection would pick out those individuals with CNVs fit to the environment.

The fact that 99.8% of Alu elements on the human genome can be found in primates indicates their functions in evolution (Deininger and Batzer, 1999). With the appearance of the nuclear membrane, introns spliced by spliceosomes, which do not exist in prokaryotes, also appear in the genome of eukaryotes. As the nuclear membrane of eukaryotes blocks communication among cells mediated by plasmids and reduces interference from the cytoplasm, stable and precise CNV extension could be mediated under control, which might be essential for the construction of the comparably complex multicellular organism. At the same time, introns might be necessary to create diversity among cells of the same individual or individuals of the same species through the insertion or deletion of gene copies. The copy numbers and expression of different genes could be distributed and oscillatory and could vary among individuals within a group to enable the group to better resist environmental factors (Figure 5). This mechanism is supported by the fact that CNVs vary and influence the expression of corresponding genes in yeast (Lauer and Avecilla, 2018).

Under the CNV extension mechanism, with the help of the nuclear membrane, genome changes in different cells could become regular and controlled. Introns could help with the production of overlapping fragments, providing enough information for CNV extension without influencing the ordinary expression of proteins too much. In multicellular creatures, introns could be used to regulate the speed of changes in gene expression, such that as the gene becomes longer with the addition of introns, its expression would change more slowly because of the longer CNV insertion required to make the change. Various genes with their different CNV extension styles, including speed, as well as the interactions between genes could enable the construction of a complex creature during embryonic development. The code inside introns might be designed to specify every insertion event. As Alu elements exist abundantly in introns and untranslated regions but are rarely seen in exons (Yulug et al., 1995; Smit, 1999), it is reasonable to think that these non-coding areas might be added to regulate the change in gene expression without interfering with the coding sequence.

The different CNVs resulting from natural selection could also be seen in highly complex species, such that the CNVs of polar bears could change rapidly according to their environment (Rinker et al., 2019). Differences in CNVs among different races and populations of humans could also be observed with a degree of selection (Sudmant et al., 2015). Moreover, CNVs are more similar within species than among related species (Nei and Rooney, 2005). In general, a range of CNVs could exist in different multicellular individuals within the same species, like in yeast, and natural selection would select those with CNVs fit to the environment. Therefore, Darwin's theory of evolution might be limited within the natural selection placed on species with distributed CNVs. It is interesting to consider a complex multicellular individual as a group of eukaryotes of one kind with different CNVs brought about by division as differentiation, which is regulated by information outside the cells, similar to natural selection.

Moreover, the insertion of an integral Alu element into a new site on the genome with extremely low probability (Deininger and Batzer, 1999), which might occur in germ line cells at the single strand breaks of genome brought about by HR between non-sister chromatids, would change the length and splicing styles of the corresponding gene slightly, resulting in a fine-tuning of gene expression and CNV extension. Besides, the new Alu insertion could influence the local CNV extension efficiency to a degree as well. It is reasonable that Alu transcription could be observed in germ line cells (Adeniyi-Jones and Zasloff, 1985; Maraia et al., 1988; Maraia, 1991). If the individual with the new Alu insertion survives, mates, and gives birth to offspring, the insertion would be passed down. Furthermore, as the stable CNVs extension of some genes is required in situations, such as embryogenesis and may need sufficient Alu transcripts, the young Alu elements might participate in with their comparably high expression. Thus, it is reasonable that most of the newly inserted integral Alu elements are those young ones (Deininger and Batzer, 1999), of which the supply exceeds demand, whereas the transcripts of other Alu elements with comparably low transcriptional level would be immediately processed once generated locally, as the demand is greater than the supply. Additionally, it might also lead to the difficulty in detecting the transcripts of those Alu elements with low transcriptional level.

In addition, the increased transcriptional level of Alu elements in mammals under stress (Liu et al., 1995) might be characteristic of primitive unicellular creatures aiming to adapt to their environment by altering their genome.

## The Extension and Contraction of CNVs Might Be Critical to Embryonic Development

About 10 years ago, it was reported that changes in CNVs could be observed in human cleavage-stage embryos (Vanneste et al., 2009). In recent years, more and more researches indicated that CNV change is common during embryogenesis and differentiation (Claycomb et al., 2004; Fischer et al., 2012, 2017; Meinhardt et al., 2015; Hannibal and Baker, 2016). Besides, CNVs of a single neuron have been found to vary widely in the human brain (McConnell et al., 2013). Moreover, differences in CNVs are found among different tissues (Henrichsen et al., 2009). These results strongly indicate the importance of CNVs in the embryonic stage. Because the change in methylation is one of the characteristics of embryonic development (Guo et al., 2014; Smith et al., 2014), and most methylation events occur at Alu elements on the genome (Khitrinskaia et al., 2003), Alu elements should be critical for embryonic development. Moreover, B1 RNA, the Alu element in mice, is expressed in fetus and germ line cells (Adeniyi-Jones and Zasloff, 1985; Maraia et al., 1988; Maraia, 1991), which also suggests a role for Alu elements in development. As changes in CNVs can be seen during embryonic development and can be related to methylation as well (Vanneste et al., 2009; Zhu et al., 2018), they might be fundamental in the construction of an individual. Therefore, CNVs representing the differentiation of the last generation have to be erased while new CNVs are written to the genome at the same time.

**Figure 6:**
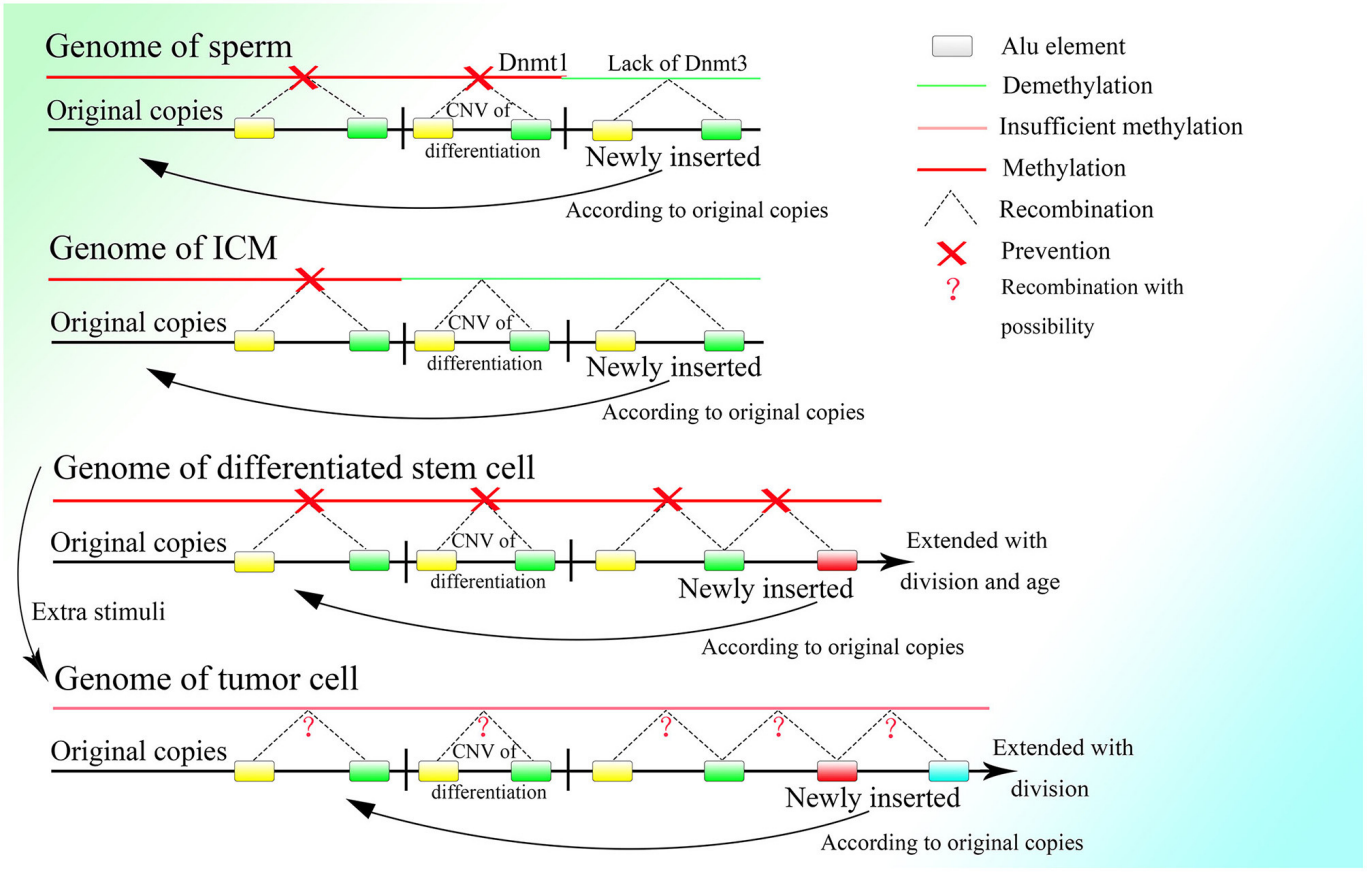
The methylation status of different kinds of cells. Demethylation under regulation can be seen in the germ line cell and embryonic cell leading to the deletion of specific regions on the genome, whereas in the somatic cell after birth, methylation is sustained at a high level and is strictly irreversible. Methylation is decreased in the tumor cell, which might bring about unpredictable HR among Alu elements and at the same time, the insertion would also occur more frequently with increased expression of corresponding genes.

Thus, it is necessary for germ line cells to avoid unwanted insertion, which would lead to CNV extension. As discussed above, the methylation level of the CGG repeats could be lower in testis tissue and sperm than in other somatic tissues in fragile X patients, with an obvious loss of CGG repeats (de Graaff et al., 1995). An experiment showed that the global genome of the sperm is highly methylated, with methylation level surpassing those of other somatic cells (Guo et al., 2014). The two results seem contradictory, but the contradiction could be explainable if some parts of the genome are hypermethylated and remain unaltered, whereas other parts are less methylated because of a lack of *de novo* methyltransferase and thus facilitate recombination among Alu elements, leading to the loss of unmethylated fragments. DNA methyltransferase 3a2 (Dnmt3a2) and DNA methyltransferase 3b (Dnmt3b), the two methyltransferases in charge of *de novo* DNA methylation, exhibit low expression in spermatogonia, and their increased expression is a signature of sperm differentiation (Shirakawa et al., 2013). Moreover, normal expression of DNA methyltransferase 1 (Dnmt1), which can copy the methylation pattern of the original strand in newly replicated DNA (Bostick et al., 2007; Sharif et al., 2007), is required for the differentiation of spermatogonia to sperm (Hirasawa et al., 2008; Shirakawa et al., 2013). It is reasonable to deduce that spermatogonia lack the ability for *de novo* methylation, which would lead to more frequent HR among Alu elements of newly inserted sequences, which would discard the unexpected CNVs extension among Alu elements. Meanwhile, Dnmt1 expression is necessary to preserve the original copies. Thus, the methylation of the original copies and the CNVs needed by the differentiation of spermatogonia and sperm, which is decided before birth, can be passed down safely and entirely during cell division in the presence of Dnmt1, whereas the DNA fragments newly inserted on the genome cannot be methylated efficiently because of the lack of Dnmt3a2 and Dnmt3b and will be deleted constantly by HR among the Alu elements with the fast proliferation of spermatogonia (Figure 6). Therefore, the hypermethylation of sperm might not be a result of the high efficiency of methylation but could be a consequence of the deletion of newly inserted fragments with a low methylation level, which is mediated by recombination among their unmethylated Alu elements.

Thus, it is reasonable that the oocyte finishes proliferating before birth and remains asleep until the individual matures with characteristic low transcriptional levels of genes (Li et al., 2018). Because fast proliferation is not necessary, the best choice to avoid unwanted insertion might be dormancy. But spermatogonia have to proliferate with high frequency, so they delete the unwanted insertion by lowering the expression of *de novo* methyltransferases, which results in the higher methylation level of the spermatogonia than the oocyte (Guo et al., 2014) and also the tumor cell (de Graaff et al., 1995). Furthermore, if too many excess fragments are inserted into the genome, it might lead to a high failure rate of embryogenesis because it is hard for the embryo to delete them completely. The fact that the nuclei of differentiated somatic cells in adult individuals possess more and longer CNVs than germ line cells might also explain the low success rate of cloning.

**Figure 7:**
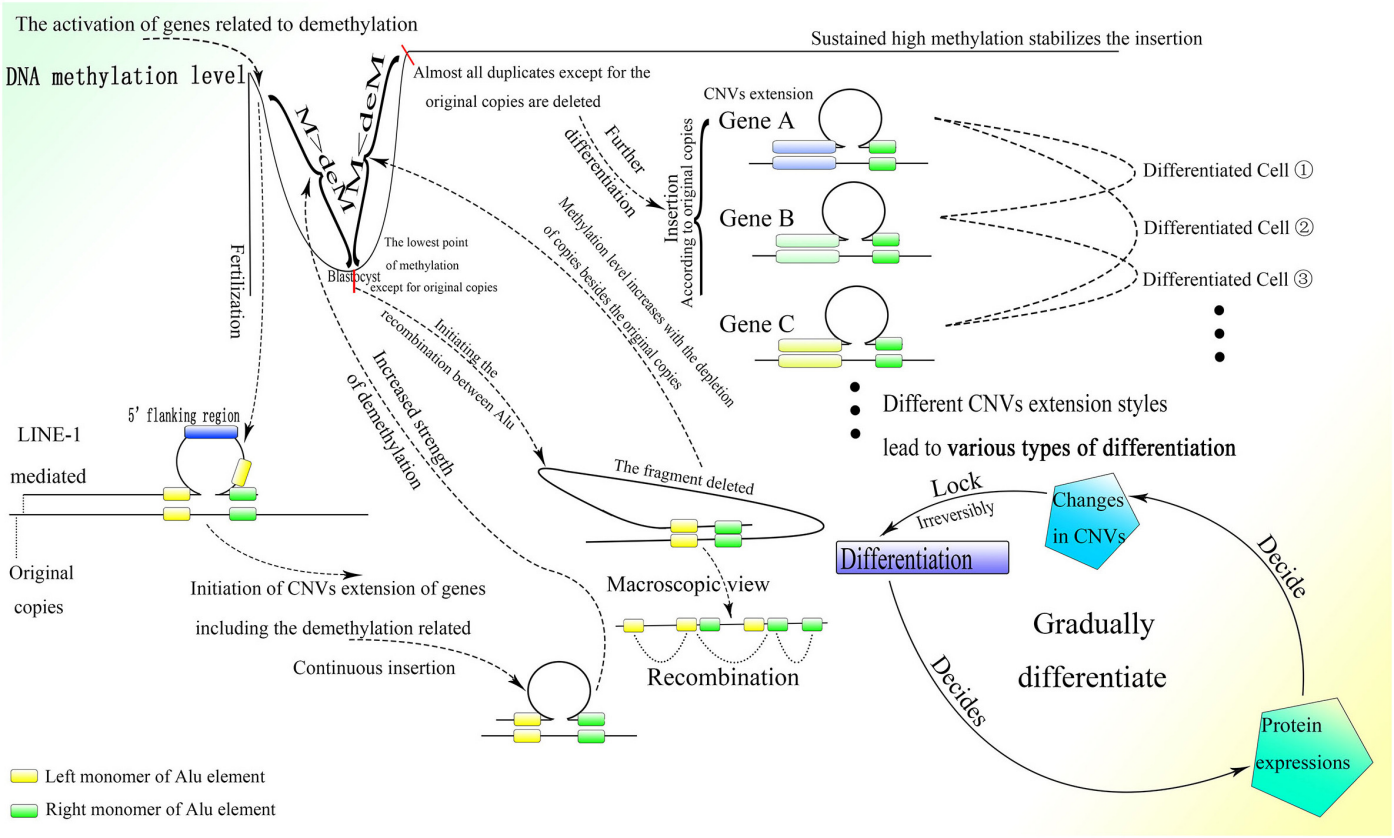
The process of embryonic development with the CNV extension mechanism. After fertilization, the CNV extension of genes related to demethylation could be initiated by LINE-1 inserting the 5′ flanking regions of these genes through 3′ transduction. Then continuous extension would be mediated by the Alu element. When the demethylation effect is sufficient to suppress the methylation effect, the Alu element would be thoroughly demethylated and HR could occur among them, leading to the deletion of inserted DNA on the genome, thus initializing cells in ICM. Then the CNVs of different genes would be extended according to the original copies of the corresponding genes. The different styles of CNVs extension in different genes would be developed next, resulting in various types of differentiation. Specifically, protein expression could influence changes in CNVs, which would irreversibly decide the differentiation, and then the differentiation could affect protein expression and lead to further differentiation. This gradual differentiation would ultimately construct an entire individual.

As described above, *de novo* methylation is reactivated after the differentiation from spermatogonia to sperm (Shirakawa et al., 2013). Regaining the ability for *de novo* methylation makes it possible to stabilize differentiation resulting from further DNA sequence insertion. Then the fertilized egg is formed by the combination of sperm and ovum, after which methylation decreases continuously (Guo et al., 2014; Smith et al., 2014). Because LINE-1 is overexpressed in the early embryonic stage (Martin, 2006), and its blocking leads to stagnation in the two-cell period (Percharde et al., 2018), it could be postulated that insertion of the 5′ flanking region of genes, which is necessary for the initiation of CNV extension, might occur after the formation of the fertilized egg and be mediated by LINE-1. LINE-1 could mediate 3′ transduction when its own polyA sequence cannot terminate transcription. Transcription would end at a stronger polyA sequence downstream and thus retrotranspose the sequence downstream to the 3′ end of LINE-1 with comparably high efficiency (Matsutani, 2006). It is interesting that two A-stretches are owned by Alu elements contain abundant adenine, which might be able to terminate transcription. Whether the presence of Alu elements can terminate the transcription of LINE-1 requires further investigation. Moreover, genome data from the NCBI GenBank showed that Alu elements that lie upstream to the 5′ flanking region of genes always lose their right parts, which can be preserved entirely in the downstream region. As the middle A-stretch of the Alu element is much shorter than the A-stretch at the tail, 3′ transduction might not be stopped by the Alu element without the right monomer but could be terminated by a full-length element. Therefore, it is reasonable to deduce that LINE-1 could mediate insertion of the 5′ flanking region of genes as well as part of its downstream sequence, which could be transcribed between the upstream left monomer of the Alu element and the entire Alu element lying downstream by 3′ transduction. Moreover, the sequences spliced by LINE-1 *in vivo* are different from those spliced *in vitro*, as LINE-1 splices various sequences *in vitro*, and the LINE-1 target sites on the genome *in vivo* show a strong preference for the TTTTAA sequence (Cost and Boeke, 1998), it is rational to deduce that the retrotransposition of LINE-1 *in vivo* requires an Alu sequence at its 3′ end. Thus, the functional retrotransposition of LINE-1 might need an Alu sequence, and at the same time that of the Alu element requires proteins produced by LINE-1. Furthermore, CNV extension initiated by LINE-1 in the early embryonic stage is also supported by reports documenting the retrotransposition of LINE-1 in germ line cells or in early embryogenesis before the germ line diverts to a distinct lineage (Ostertag et al., 2002; Prak et al., 2003). Moreover, LINE-1 retrotransposition that occurs during the embryonic stage can also increase gene expression and affect differentiation (Muotri et al., 2005). Alu elements upstream to the 5′ flanking region without the right monomer could facilitate 3′ transduction with less A-stretch, and at the same time asymmetric Alu construction of 3′ transduction transcripts would increase the efficiency of correct combination to the insertion sites. By the way, as LINE-1 is rich in splice sites (Belancio et al., 2006), all or part of the LINE-1 sequence on the 5′ part of the transcript might be discarded, leaving only the 3′ transduction part to insert. Furthermore, the multiple polyA on LINE-1 could lead to premature polyadenylation and thus truncation of full-length transcripts (Perepelitsa-Belancio and Deininger, 2003), which might result in the requirement for a high transcriptional level of LINE-1 to produce enough mRNA with 3′ transduction. After the initiation of CNV extension by LINE-1, the following sequence would be inserted between the two parts of the Alu element newly inserted downstream to the 5′ flanking region with the help of the corresponding lariat, and then the CNVs would be extended continuously with guidance from the right monomer of the Alu element (Figure 7).

As proteins, such as activation-induced cytidine deaminase and histone lysine demethylase (KDM) could affect demethylation, and the knockdown of KDM7A could seriously influence the development of inner cell mass (ICM) (Rissi and Glanzner, 2019), the genes whose CNVs are extended at the beginning of embryogenesis might be related to demethylation. The continuous CNV extension of these genes with the demethylation effect increases their expression, which antagonizes methylation. In addition, the promoted transcriptional level of LINE-1 in the early embryonic stage might also be a result of demethylation, as methylation could largely inhibit its activity (Woodcock et al., 1997). With the splitting of the fertilized egg, the whole genome except for the original copies with extra protection would be demethylated gradually as the cell divides, and ultimately the methylation level would hit rock bottom before implantation (Guo et al., 2014; Smith et al., 2014), which is sufficient to conduct HR among Alu elements and thus discard the fragments among them. Ultimately, almost all inserted CNVs remaining on the genome would be deleted and there would remain only the original copies of these CNVs and those fundamental genes that could not be extended or deleted (Figures 6, 7). This process is supported by the fact that *de novo* methylation can still be observed during the demethylation period in the early embryonic stage (Zhu et al., 2018). Moreover, it coincides with the fact that some CNVs in the hypomethylated embryo increase, whereas CNVs in the hypermethylated embryo decrease before implantation (Zhu et al., 2018). In addition, the chromosome instability as frequent segmental deletions, duplications, and amplifications and the change in CNVs found in human cleavage-stage embryos (Vanneste et al., 2009) demonstrate the insertion and deletion of fragments on the genome as well. Note that the fact that changes in CNVs can be observed directly only in the embryo might be due to the large change in CNVs within a short time; change would be much smaller and slower after birth and thus more difficult to detect. Because the methylation level of ICM is significantly higher than that of trophectoderm (Zhu et al., 2018), ICM could not differentiate into trophectoderm, perhaps because trophectoderm is differentiated from the ICM of the last generation. This is also consistent with the differences in gene expression between ICM and trophectoderm (Ozawa et al., 2012). Then the methylation level of the genome would increase sharply (Guo et al., 2014; Smith et al., 2014), which could be a result of the deletion of less methylated CNVs on the genome mediated by HR among demethylated Alu elements. The same kind of Alu element clustered in corresponding areas in human chromosomes (Filatov et al., 1987; Grover et al., 2004) makes possible continuous HR in the ICM, which could almost completely erase the CNVs inserted.

This mechanism is also supported by results showing that LINEs are generally highly methylated in sperm but demethylated in the early embryo, with partial remethylation in human embryonic stem cells and complete hypermethylation in somatic cells (Smith et al., 2014). Note that the methylation of short interspersed elements, such as Alu elements diminishes rapidly to nearly complete hypomethylation over preimplantation and is uniformly regulated regardless of subfamily (Smith et al., 2014), which suggests the possibility of HR among Alu elements and the deletion of CNVs on the genome as well. Moreover, expression of proteins related to methylation, such as Dnmt3a, Dnmt3b, Dnmt3l, methyl-CpG binding domain protein 2, and methyl-CpG binding protein 2, is increased between 2- and 12-fold from ICM to embryonic stem cells (Tang et al., 2010), which also demonstrates HR among Alu elements with insufficient methylation in ICM and further CNV extension with sufficient methylation in embryonic stem cells. Moreover, expression of c-Myc, a well-known reprogramming factor, and some other genes is highly heterogeneous in ICM but becomes homogenous in embryonic stem cells (Tang et al., 2010), which provides evidence of the initialization of CNVs on the genome in ICM. With most of the inserted CNVs deleted, all cells within ICM become almost equal, with similar expression styles (Tang et al., 2010), and are able to further differentiate into almost all kinds of cells (Das et al., 2008) depending on the different CNV extension results.

As most CNVs are deleted, including those of genes associated with demethylation, the effects of methylation would surpass those of demethylation, again providing cells with the *de novo* methylation ability to stabilize further CNV extension on the genome. At the same time, the proliferation that occurs in the peri-implantation period produces lots of embryo cells, of which the genomes are almost empty except for the original copies, laying foundation for further differentiation depending on protein expression styles and levels, which are decided by further CNVs extension.

Thereafter, the high methylation in embryonic somatic cells could be sustained by the markedly increased expression of corresponding genes until birth (Tang et al., 2010; Smith et al., 2014; Guo et al., 2015). As DNA methylation could prevent the occurrence of HR (Colot et al., 1999) and thus inhibit the deletion brought about by Alu elements, the unique styles of CNVs extension in different cells could lead to different types of differentiation and would be irreversible throughout one's life. Moreover, differentiation would influence protein expression, which could affect further differentiation with CNVs extension. Through this cycle, an entire individual with various types of differentiation could be gradually constructed (Figure 7). The fact that unique protein expression could be observed in different tissues and at different times during embryonic development (Tang et al., 2010) coincides with this process to a degree. Besides, the facts that the production of extrachromosomal circular DNA changes with developmental stage in frog and flies (Cohen et al., 1999, 2003) also indicate that the genome receives various reconstitution during different embryonic stages.

**Figure 8:**
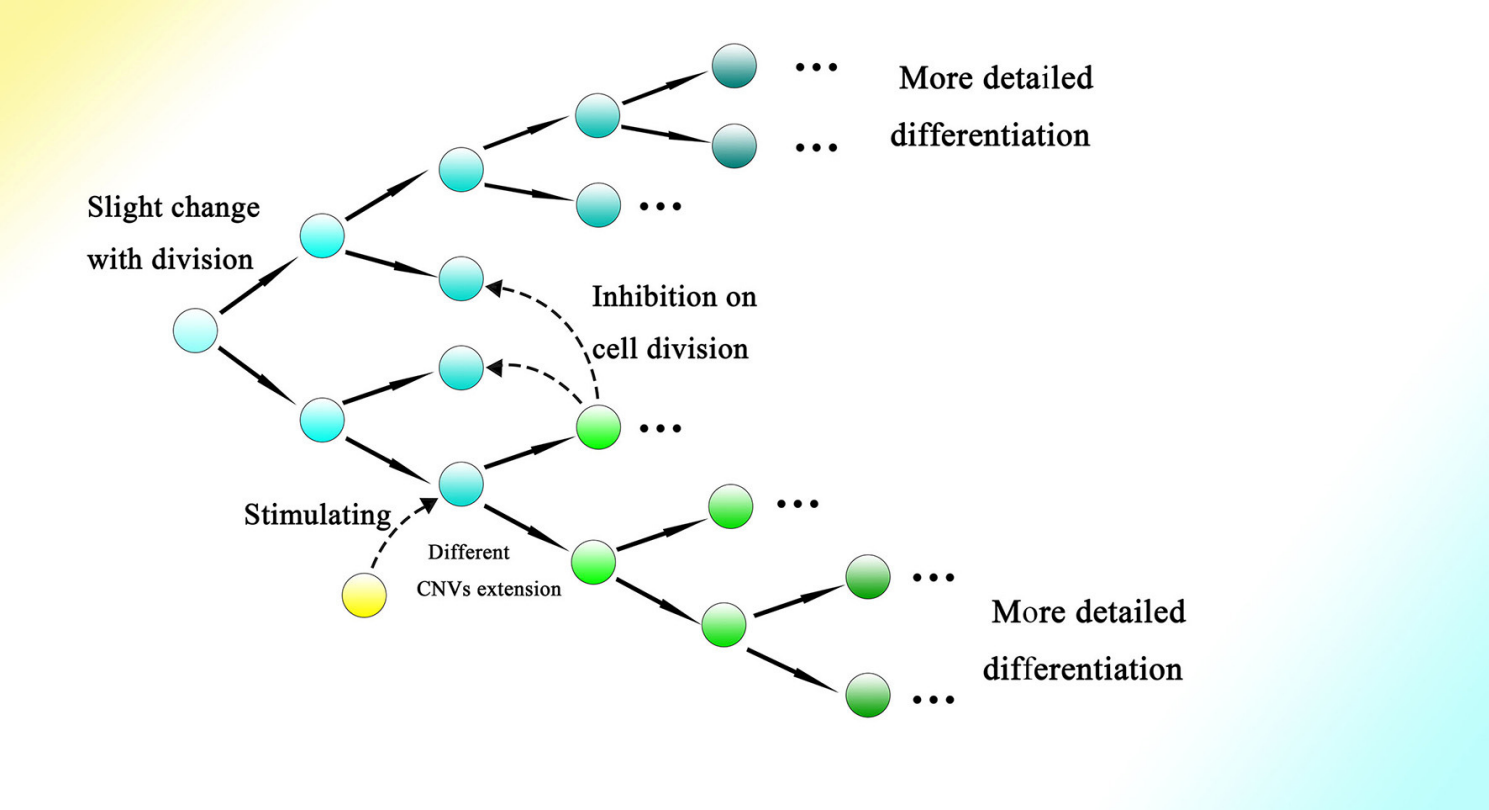
Cell division could bring about gradual genome change and thus differentiation during embryogenesis. The genome of the cell would be changed slightly with every division. The occurrence of division and the direction of differentiation are affected by adjacent cells or other external effects. The gene expression of cells could be influenced by adjacent cells, resulting in changes in CNVs extension style and therefore an altered direction of differentiation. Finally, as the cell divides, detailed and intricate differentiation would be achieved.

Moreover, symmetrical development of the embryo might indicate the relationship between differentiation and cell division to a degree. As every split of the cell could generate relative changes, as the cell divides more information on the genome would gradually be read through CNVs extension, contributing to more intricate and detailed differentiation. Meanwhile, interactions among cells and other outside effects could also influence the occurrence of division and the direction of differentiation by affecting the gene expression of the corresponding cell. Therefore, the process of embryogenesis might be reducible to permitted cell divisions with conditions to generate changes during splitting (Figure 8).

In addition, with the help of high methylation, information about differentiation (the CNVs extension style) could be written firmly into the genome. The length and expression of a gene as well as the transcriptional level of Alu elements within it could decide its extension rhythm. Furthermore, the high transcriptional level of corresponding genes or elevated transcription of transposons within them (Adeniyi-Jones and Zasloff, 1985; Maraia et al., 1988; Maraia, 1991) could make possible firm CNV extension with every cell division. Moreover, the combination of different CNVs extension of different genes could create great variation to construct an entire individual, with different kinds of cells and their corresponding functions. From the facts that the expression of specific transcription factors can decide differentiation (Takahashi and Yamanaka, 2006; Hamazaki et al., 2021), we infer that the embryogenesis might consist of different stages with distinct expression patterns of transcription factors and change in CNVs might play a critical role in the activation and inactivation of genes of various transcription factors. The order of the CNVs changes rather than the final CNVs might contribute more to the differentiation and embryogenesis. In another word, the process could be more important than the result. As the final CNVs could be similar among different kinds of differentiated cells, it is easy to ignore the intermediate process.

In addition, the reason why postulated changes in the number of methylated and demethylated sites are not detected might be because of the difficulty of observing all CpG sites on the global genome (Chatterjee et al., 2013; Guo et al., 2013).

We may exclude other possibilities for CNVs changes during embryogenesis. As for the decrease in CNVs, because dsDNA breakage is dangerous for the preservation of the genome, frequent dsDNA breakage during embryogenesis is irrational and would be obviated. Thus, the only possible way to decrease CNVs is through HR, which would not cause dsDNA breakage. It is reasonable to deduce that HR is mediated by Alu elements given the demethylation of Alu elements and the characteristic change in methylation during embryogenesis. With regard to the increase in CNVs, templates are needed for CNVs extension. There are two possible templates: the genome and RNA transcripts. We can exclude the possibility of the genome, as dsDNA breakage is dangerous and unregulated and it is hard to exert complex function by genome interaction, while HR could only exchange fragments and would not lead to an increase. Accordingly, using RNA transcripts as templates to mediate HR is most likely to result in an increase in CNVs. Moreover, the extension should be guided by Alu, which would make it convenient for further Alu-mediated HR to delete all inserted fragments.

## The Dynamic Change in CNVs Might Be Associated with the Development of Senescence

A contradiction exists in present knowledge of senescence in differentiated somatic cells and stem cells. Although it is believed that telomerase can preserve the length of telomeres in adults, and telomeres become shorter over time in most human somatic cells as cells divide (Harley et al., 1990; Chang and Harley, 1995; Sitte et al., 1998), this does not align with evidence showing that only in germ line cell and adult stem cell populations the activity of telomerase is sufficient to sustain the telomere length (TL) of homeostasis (Lee et al., 1998; Herrera et al., 1999; Dokal, 2000; Hao et al., 2005; Liu et al., 2007), and in most fully differentiated cells expression of telomerase is virtually undetectable (Meyerson et al., 1997). As a somatic cell without telomerase activity will continuously lose 50–200 bp telomeric DNA with every division (Kaminker et al., 2001; Halytskiy, 2009; Mathew et al., 2010), and the full length of the telomere in humans is only about 10,000 bases (Akkad et al., 2006; Invernizzi et al., 2014), it is definitely not sufficient to maintain normal functioning and vitality of somatic cells to the death of an individual. Although some kinds of cells can extend TL without the aid of telomerase through alternative lengthening of telomeres (ALT), ALT-positive cells can only be found among tumor cells (Shay et al., 2012).

**Figure 9:**
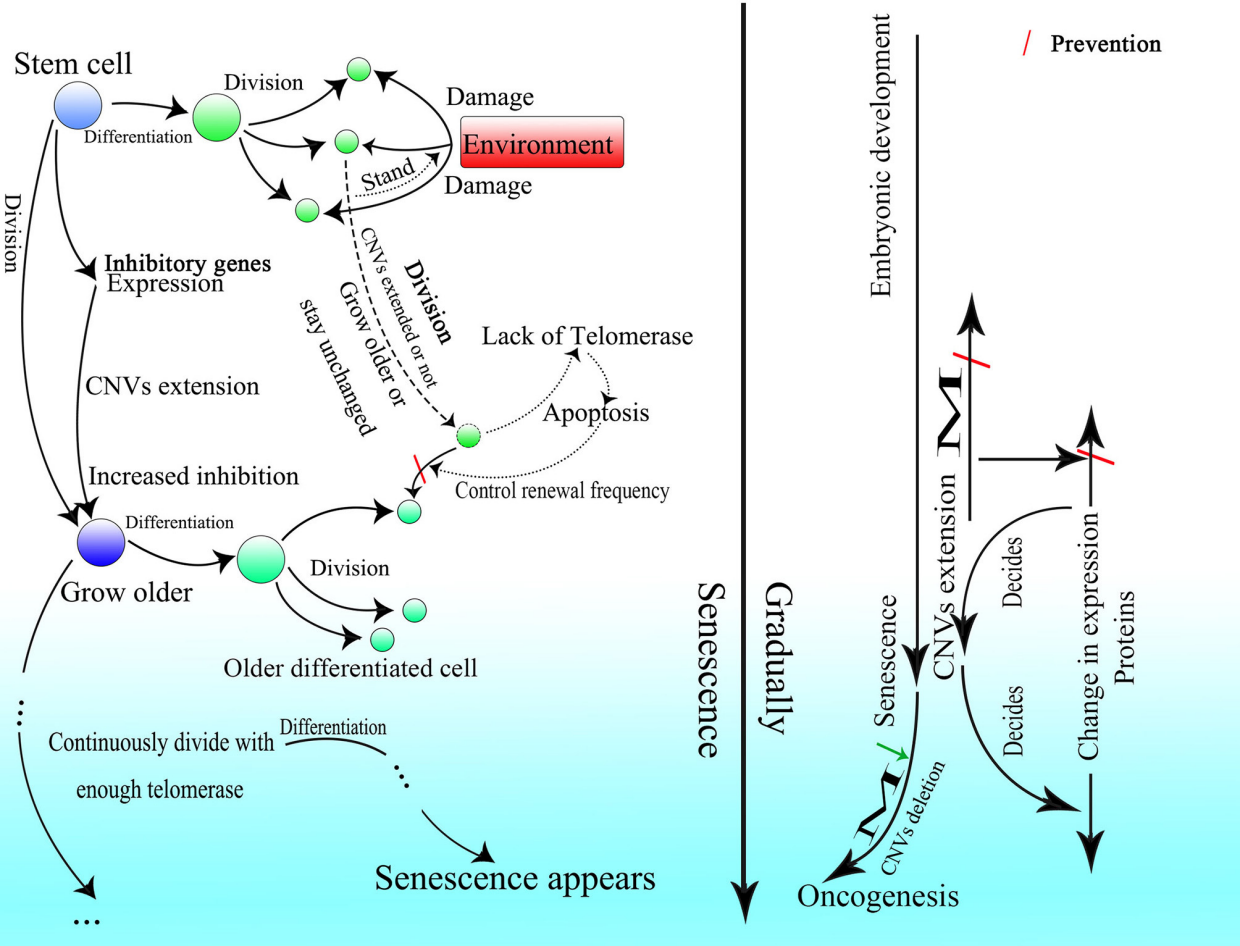
A proposed mechanism of senescence under the CNV extension mechanism. The entirely differentiated cell could be differentiated from the stem cell, perform certain functions, and protect the stem cell lying inside. The CNVs of inhibitory genes would be extended as the cell divides and gradually lead to senescence in the stem cell. The stem cell could continuously divide with sufficient telomerase activity and ultimately lead to senescence as the cell divides. Because of a lack of telomerase, the somatic cells would be forced to apoptosis and then substituted by older cells differentiated from stem cells. Moreover, methylation would have critical effects in terms of stabilizing the genome from embryogenesis to senescence.

**Figure 10:**
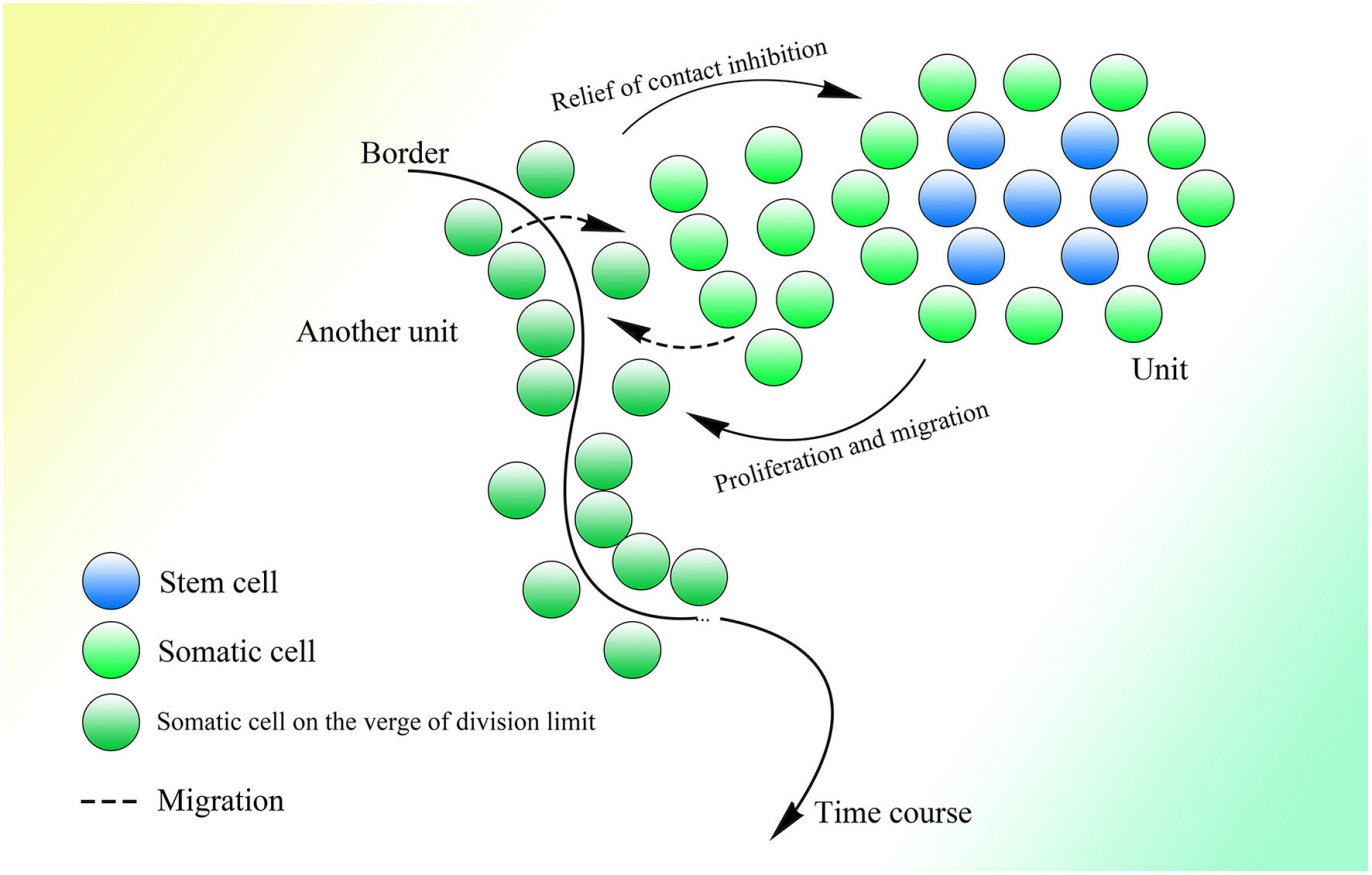
A possible pattern in which cell proliferation could be strictly regulated. According to tumor stem cells and their proliferation pattern, tissue could be divided into lots of units that would consist of stem cells and somatic cells. The central stem cells would be surrounded by other stem cells, and they would not differentiate and could only divide into stem cells; the stem cells near the somatic cells would receive stimulation from stem cells and gradually differentiate to somatic cells. The proliferation of cells at the border between the two units might fluctuate. When the cell density of one side decreases, the cells below the border and those on the other side of the border will migrate toward it, resulting in increased cell density on this side of the border and decreased cell density on the other side of the border and thus stimulating the cells below the border of the same side and on the other side of the border to proliferate. As the cells on the other side of the border are on the verge of the division limit, further division will lead to apoptosis and decreased cell density. This cycle will ensure that all cells divide periodically.

The transcription of LINE-1 can be detected in adult stem cells and normal human tissues (Belancio et al., 2010), which indicates that the change in CNVs might exert its effects after birth. Therefore, a mechanism for telomeres and senescence based on CNV extension is proposed. There might exist a group of genes that are critical for the stable work of cells with inhibitory effects and comparably high expression of themselves or the Alu elements within them, which could extend their corresponding CNVs steadily as cells divide. Note that CNV extension could only happen during cell division, and thus senescence would be closely related to the times the cell divides. With the cell dividing and CNVs being extended, inhibition from these genes taking charge of cell activity would increase gradually, finally leading to senescence. The differentiated stem cells in different tissues own the activity of the telomerase with which the telomere could be extended consistently, and the telomerase activity is inhibited via differentiation and thus absent in entirely differentiated somatic cells, limiting the times they could divide. These entirely differentiated cells could protect the stem cells lying deeply and exert their differentiated functions. As the entirely differentiated somatic cells could be consistently differentiated from the stem cells, their division could largely reduce the number of times the stem cells need to divide and therefore slow the aging of the stem cells. Although the entirely differentiated somatic cells might also gradually become older, the lack of telomerase would bring them to apoptosis before distinguishable differences with the corresponding stem cells could be seen (Figure 9). The positive correlation between the expression of most genes, especially oncogenes and tumor suppressor genes, and their corresponding CNVs (Shao et al., 2019) provides evidence of the mechanism. Moreover, the reduced copy number of *BRCA1*, a tumor suppressor gene, can be observed in breast cancer (Wei et al., 2005), which indicates the importance of inhibitory genes' CNVs in maintaining the ordinary functioning of the organism. It is rational to think that if insufficient inhibition could lead to oncogenesis, then sufficient inhibition would result in senescence conversely. In addition, the genome alteration generated from cell division also coincides with the transformation of gene expression and status exhibited in cultured cells after passage. As cell proliferation can be inhibited by contact, and the cell tends to migrate toward places with lower cell density, cell division can be regulated accurately, and thus senescence would be strictly controlled (Figure 10). Migration would convert to invasion during oncogenesis.

Moreover, the fact that TL varies in different tissues and is shortest in whole blood (Demanelis et al., 2020) demonstrates the close relationship between TL and the renewal frequency of different tissues. As the transcription of LINE-1 might be deficient in some kinds of somatic cells except for adult stem cells (Belancio et al., 2010), which could result in a lack of producers and carriers of ORF2p, there is also a possibility that the CNVs of the entirely differentiated somatic cells could not change and that these cells would not become senescent; therefore, TL in these cells could only be used to decide their renewal frequency and thus keep pace with the senescence of the corresponding stem cells. Although these entirely differentiated somatic cells could not become senescent, they would be forced to apoptosis and substituted by slightly older cells with restored TL divided from stem cells growing slightly older (Figure 9). The multicellular organism might exist similar to the tumor, in which only very few tumor cells have the ability to proliferate constantly (Park et al., 1971; Bonnet and Dick, 1997) and the growth of whole tumor depends largely on them.

Moreover, the fact that the TL of testis is the longest and would not change with age could also suggest that TL might not correlate directly with senescence (Demanelis et al., 2020). But the average length of telomeres in cells decreases with age (Cherif et al., 2003), which could be a result of senescence in differentiated stem cells that telomerase activity in them is inhibited by the increased expression of inhibitory genes with age and thus leads to decreased TL. This mechanism might be partly consistent with the finding that epigenetic aging is not closely related to telomerase expression (Kabacik et al., 2018). From our perspective, detection about TL on the different kinds of single cell from the same tissue is recommended.

It is interesting that the mechanisms related to telomeres and telomerase are similar to the CNV extension mechanism in that both are associated with reverse transcription, recombination, and cell division (Shampay and Blackburn, 1988; Sobinoff and Pickett, 2020). Furthermore, some telomere extension can also correlate with the transposon (Danilevskaya et al., 1997; Villasante et al., 2008).

## The Effects of Dynamically Changing CNVs on Oncogenesis

The fact that women with the breast cancer 1/2 (*BRCA1/2*) mutation, which might be inborn, have a high risk for breast cancer onset at a certain age with high probability (Kuchenbaecker et al., 2017; Copson et al., 2018) strongly indicates an accumulative effect in terms of oncogenesis. Furthermore, the expression of most genes, especially oncogenes and tumor suppressor genes, is positively correlated with their corresponding CNVs (Shao et al., 2019), which suggests that CNVs might play an important role in oncogenesis.

In most cases, a central neurocytoma occurs within the ventricles (Bonney et al., 2015), given the existence of the multipotent stem cell-like population in the subventricular region of the brain (Gritti et al., 1999), which indicates that tumor cells might develop from stem cells. In fact, we rarely see tumors develop from entirely differentiated mature neurons. Moreover, because tumor cells have the same telomerase activity as stem cells, most tumor cells might originate from differentiated stem cells of different tissues. The fact that only very few cells called tumor stem cells, which are separated from tumors, are enable to form colonies *in vivo* and *in vitro* (Bruce and Van Der Gaag, 1963; Bergsagel and Valeriote, 1968; Park et al., 1971; Fidler and Kripke, 1977; Hamburger and Salmon, 1977; Fidler and Hart, 1982; Bonnet and Dick, 1997) supports the deduction.

Given the characteristics of fast proliferation and high metabolism, it seems that the inhibitory genes exerting effects on developing senescence are overcome by genes of stimulating effects, such as oncogenes. Therefore, stem cells, whose inhibitory genes are largely inhibited by stimulating genes with abnormally increased CNVs resulting from a high transcriptional level, might develop into tumor cells, with telomerase activity. Specifically, tumor cells might originate in one of two ways.

First, with the CNVs of oncogenes extended, the oncogenes could gradually antagonize the inhibitory genes, which would ultimately lead to oncogenesis. In normal stem cells, stimulating genes are extended relatively slowly because of the comparatively low expression of these genes or the Alu elements within them. But when external or internal stimuli, such as inflammation exist, expression of these stimulating genes can become elevated, and thus the CNV extension efficiency of these genes can increase as well. Most of the time, it is still relatively safe when there is no mutation within these genes. But in the presence of inborn or acquired mutations, stimulating genes with abnormally extended CNVs could exert stronger inhibitory effects than those without mutations on their opposite genes and ultimately suppress them. As the expression of inhibitory genes is inhibited during oncogenesis, these genes would gradually become hypermethylated (Issa et al., 2001). At the same time, the increased inhibition of the expression of inhibitory genes could also block their normal CNV extension. Finally, the balance between the stimulating genes and inhibitory genes would be completely disrupted, and the tumor cell would be born. There definitely exists a certain relationship between chronic inflammation and cancer (Kuper et al., 2000; Affara and Coussens, 2007; Murata, 2018; Singh et al., 2019). Moreover, this mechanism is also supported by the markedly increased risk for breast cancer in persons with *BRCA1/2* gene mutations at a range of ages (Armes et al., 1999). The fact that the genes *C-myc* and *c-erbB-2* are amplified in some human breast cancers and that the degree and frequency of the increased amplification are associated with advanced clinical stages of the disease (Slamon, 1990; Lovekin et al., 1991; O'Reilly et al., 1991; Paterson et al., 1991) supports this mechanism as well. Furthermore, the copy number changes in *c-erbB-2* are much more specific in breast cancers than in gastric cancers (Niu et al., 2020), which also indicates that changes in CNVs play a critical role in oncogenesis.

In addition, if the inhibition from the inhibitory genes weakens, oncogenesis would also occur. For example, the mutation of *BRCA1*, a tumor suppressor gene, could result in its dysfunction and elevate the risk of developing breast cancer at a certain age (Armes et al., 1999). In the beginning, the gap between the normal *BRCA1* and the one with the mutation is not obvious, but as their CNVs extend, this gap widens and the *BRCA1* with the mutation cannot provide sufficient inhibition to suppress the oncogenes, which ultimately leads to oncogenesis. The *BRCA1* promoter is methylated while the *BRCA1* copy number is reduced in sporadic breast cancer (Wei et al., 2005). Moreover, many Alu elements distributed on BRCA1 indicate that the CNV of *BRCA1* could be extended via CNV extension (Wang et al., 2019).

Also, the HR that occurs among Alu elements of inhibitory genes with insufficient methylation could also lead to oncogenesis. As the methylation of Alu elements decreases with age (Doshi et al., 2004; Bollati et al., 2009), the frequency of recombination increases (Figure 6). When recombination occurs on inhibitory genes, their expression decreases markedly. Meanwhile, the inhibition of stimulating genes by inhibitory genes would ease, disrupting the balance between them. Then the CNVs of the stimulating genes could be extended with increased efficiency because of their elevated expression, which would ultimately lead to the development of cancers.

Furthermore, the recurrence of glioma after excision is often accompanied by an elevated grade (Jaeckle et al., 2011), which might be the result of further CNVs extension of stimulating genes responsible for oncogenesis with the cell proliferation after excision.

Moreover, the transcription of LINE-1 along with its expression of proteins required for Alu elements to function is increased in tumor cells (Su et al., 2007; Harris et al., 2010), and the markedly decreased methylation of Alu elements is also observed in tumor cells (Cho et al., 2007), which could emphasize the importance of retrotransposons in oncogenesis.

The genes that take charge of the main structure of the cell always lack Alu elements (Grover et al., 2003), which might be one reason why tumor cells cannot destroy themselves when their genes are compromised by abnormal DNA insertion and deletion (Liu and Mi, 2019). It seems that cells of multicellular organisms are controlled and chained by inhibitory genes, but tumor cells get rid of them by accident and thus exhibit characteristics of unicellular organisms.

Even though it is hard to observe how a normal cell converts into a tumor cell *in vivo*, we can still investigate the differences in CNVs between tumor cells and corresponding advanced tumor cells, which always reappear after tumor excision. A correlation between the changes in CNVs and advanced cancer would suggest a role of CNVs extension in oncogenesis.

## Associations with Other Diseases

Similarly, if the expression of genes related to metabolism is abnormally increased, their CNVs extension efficiency could also be promoted, leading to irreversible metabolic disorder, such as type II diabetes mellitus, which includes obesity as a risk factor and cannot be cured completely (Afridi and Alam, 2004; Han et al., 2006). Moreover, the Prospective Urban Rural Epidemiological study showed that high consumption of white rice can result in an increased risk for incident diabetes (Hu et al., 2012; Bhavadharini and Mohan, 2020). Moreover, the result of a recent study that nucleotide reverse transcriptase inhibitors can improve insulin sensitivity and reduce the development of type 2 diabetes supports the relationship between the development of type II diabetes mellitus and CNVs extension as well (Ambati et al., 2020).

The CNV extension mechanism might also be involved in viral infection. The transcription of Alu elements is elevated when cells are infected by a virus (Jang and Latchman, 1989; Russanova et al., 1995). Moreover, integrations of HIV-1 peak in the right part of Alu elements on the genome while they are almost equal in other places (Cohn et al., 2015). Under the CNV extension mechanism, when a gene on the human genome inserted by the HIV-1 genome is transcribed, the whole HIV-1 genome could also be transcribed at the same time, which would produce overlapping lariats containing different parts of the HIV-1 genome. The initial integration of HIV-1 into the human genome occurs on its own, resulting in integration on the genome with little selection. But as for those integrating into the right part of an Alu element, the branch point of lariats could originate from the virus genome and attack the sequence upstream as the left part of the Alu element. Then the lariat produced would be linked with the right monomer of the Alu element as usual, ultimately generating lariat-Alu dsDNA with the left and right parts of the Alu element on the two flanks and part of the right monomer of the Alu element as well as the 5′ region of the virus genome between them, which could insert the 5′ sequence of the virus into another right part of the Alu element on the genome. Then partially overlapping lariats would be produced consistently and gradually complete the insertion of a clone of the HIV-1 genome at another Alu element on the human genome (Figure 2).

Furthermore, quantities of expanded clones originate from a few clonally expanded cells (Maldarelli et al., 2014; Wagner et al., 2014), and these cells with a persistent ability to proliferate might be classified as the stem cells described above with the ability of CNVs extension. Moreover, almost all expanded clones of HIV are defective (Cohn et al., 2015), which indicates that expanded clones are not the result of ordinary integration of HIV. Furthermore, there are much more defective clonal integrations into or near the Alu elements within highly expressed genes than those intact single integrations (Cohn et al., 2015), implying the defective expanded clones are integrated by distinct mechanism. The fact that expanded clones are defective suggests that they originate from the lariats, which are produced by integral HIV genome transcripts and only carry part of the genome.

Lariats with the 5′ end falling in Alu elements cannot ordinarily be produced absolutely, but the insertion of a virus genome introduces sequences, such as its long terminal repeats that do not exist in the human genome, leading to incorrect recognition of the spliceosome. Thus, with the help of the CNV extension mechanism, insertions of the HIV-1 genome would occur more frequently in the right part of Alu elements on the genome, as reported previously (Cohn et al., 2015). In addition, the CNV extension system could be occupied by the virus integration, thereby interfering with the ordinary extension of CNVs on the human genome, which might facilitate oncogenesis.

## Back to Neurodegenerative Diseases

**Figure 11:**
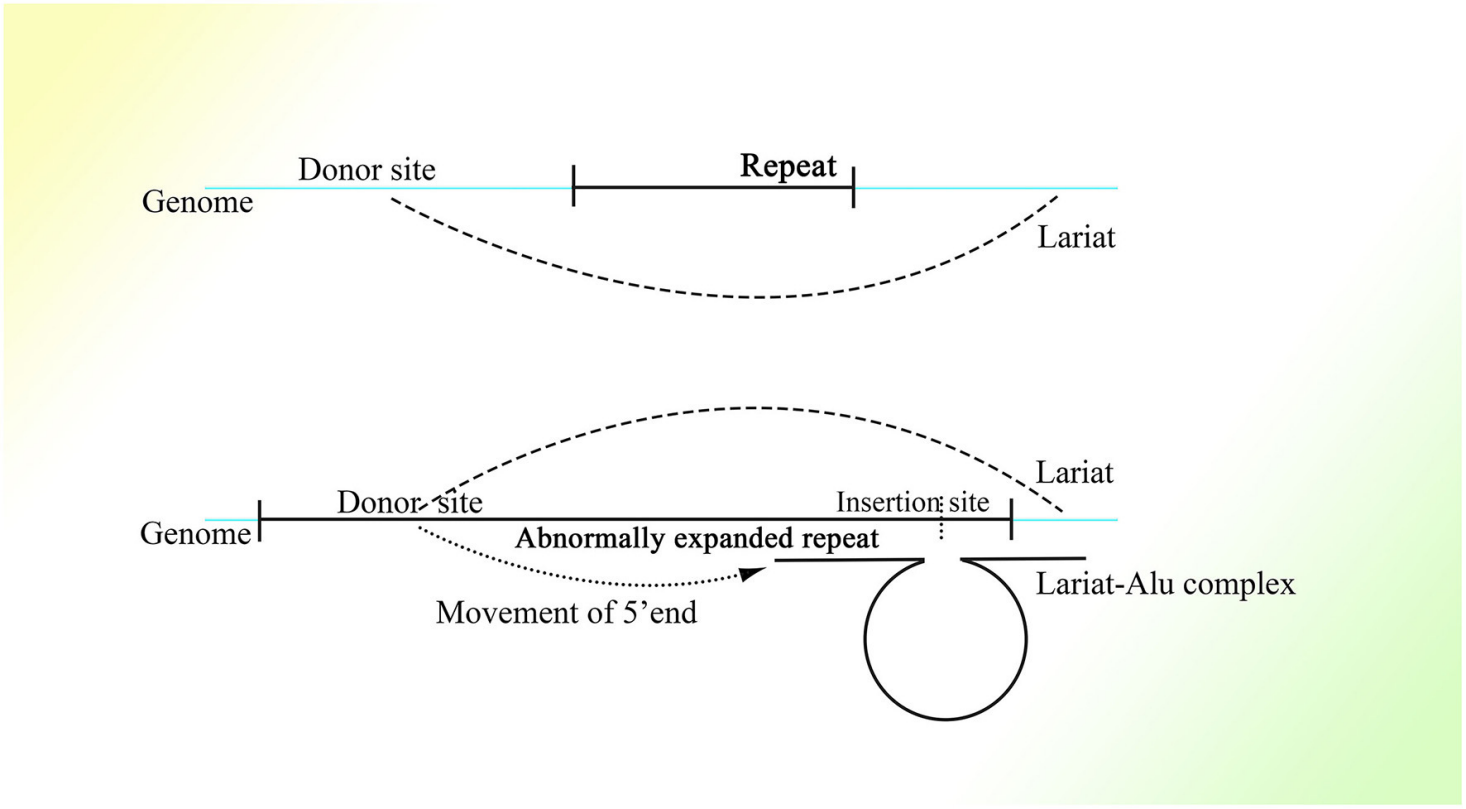
A possible mechanism explaining expansion of the repeat sequence in HD and FXS affected by transcriptional level. As the abnormally expanded repeat is too long to jump across to produce a normal lariat, the 5′ end of the lariat could fall into the repeat sequence, which would ordinarily not happen. The repeat sequence could obscure recognition of the left foot of Ω, making its complementary combination to the repeat downstream, which would create an insertion site that should not exist. This would lead to the insertion of the repeat sequence, and thus the repeat could be expanded.

According to the CNV extension mechanism, lariats produced by abnormal splicing would be necessary for the repeat expansion in neurodegenerative diseases, such as HD. It is interesting that both the CAG repeats in HD and the CGG repeats in FXS contain the GC binucleotide, which could be recognized as a donor site. Trinucleotide repeats are ordinarily short, and thus splicing could happen over the repeats, avoiding abnormal extension. But when the repeats are too long to jump over, the branch point will attack the wrong donor sites within repeats, obscuring recognition of the sequence during insertion from the 3′ end of both lariat-Alu ssDNA and one of the strands of lariat-Alu dsDNA, of which the sequence should be specific but changed to repeat sequence in HD and FXS, ultimately leading to the abnormal repeat expansion (Figure 11). Moreover, although recombination between two parts of trinucleotide repeats of sufficient length might occur and thereby reduce the length of the repeats, the decrease would be more than offset by the increase and ultimately result in the onset of HD. In addition, as the CGG repeat required for normal functioning of *FMR1* is still too long to jump over, the AGG codon within the repeats might act as a foothold for the branch point to attack to prevent it from falling into the repeats. Meanwhile, the AGG codon could also serve as a mark of recognition during the insertion, thus preventing abnormal repeat expansion.

## Discussion

The success of the induced pluripotent stem cell (Takahashi and Yamanaka, 2006) suggests that it is the expression style of proteins in the cell that decides what kind of cell it should be. According to this point of view, the genome is like a chain limiting the expression styles of proteins. Differences in protein expression styles exist among cells from different tissues and between young and old cells, which suggests that their genomes could also be different. Although epigenetics might be able to explain part of it, a mechanism for the initiation of these changes is still lacking. In this article, we presented a possible model with partially overlapping lariats originating from pre-mRNA splicing and retrotransposons on the genome and argued that CNVs of genes might be changing all the time with cell division during embryonic development, senescence, and oncogenesis. Moreover, CNVs could also play an important role in evolution. With this mechanism proposed, many confusing puzzles in these areas could be explained.

**Figure 12:**
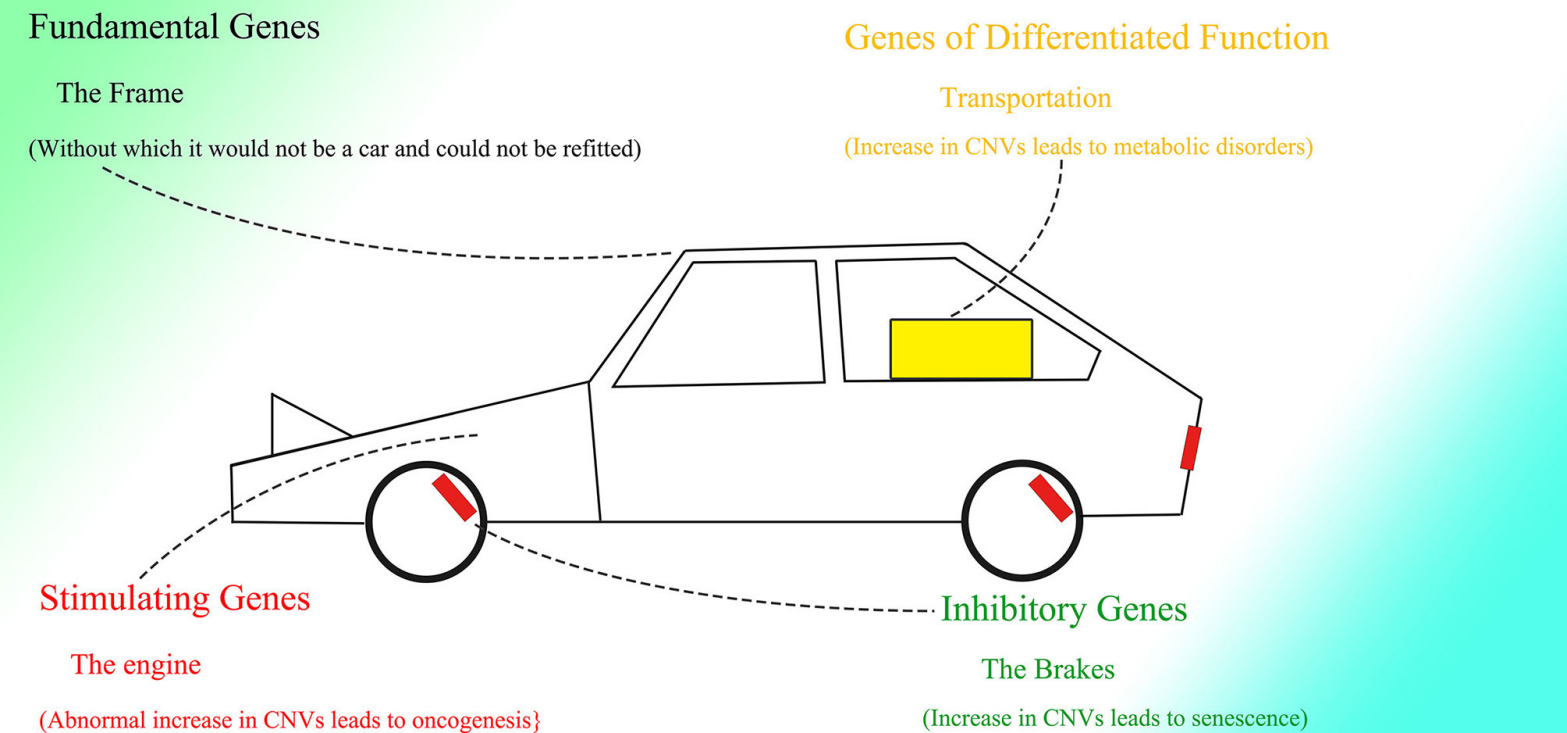
The entire genome might be compared to a car. The frame (the fundamental genes) cannot be altered. A modified engine (increased CNVs of stimulating genes) results in increased speed (oncogenesis). The brakes (inhibitory genes) slow the car down (cause the organism to become senile). The genes of differentiated functions can also be modified.

Because Alu elements are more abundant in genes involved in metabolism, transport, and signaling processes and much less common in genes related to information pathway components and structural proteins (Grover et al., 2003), the core functions sustaining the fundamental activities of cells might not be changed by the CNV extension mechanism, just as the main structure of a car, such as the frame (genes of structural proteins) and drive system (genes of information pathway components). At the same time, the car could be changed by adding different engines (such as stimulating genes) to speed up and brakes (such as inhibitory genes) to slow down. Moreover, it could also be modified by adding other functions that are not fundamental, such as functions brought about by differentiation (such as genes associated with metabolism or functions derived from differentiation). During the embryonic period, the car accelerates, and between birth and senescence its speed is gradually decreased by the brakes. All of these processes should be precisely regulated; if the car goes too fast, a tumor cell could develop (Figure 12). In addition, different species could be compared to different cars with different parts. Moreover, Alu elements are similar to the building blocks on the genome with which a variety of structures can be constructed; the genome can also be disassembled by these building blocks, but methylation is the glue preventing the structure from being disassembled. The irreversible methylation sustained throughout one's life ensures normal and stable functioning of various differentiated cells and stabilizes CNVs extension, which gradually leads to senescence; demethylation can result in initialization in the embryo and disorder in tumors.

In general, people pay more attention to the main body of an object and ignore things thought to be less important, whose effects are often secret and localized. After the Human Genome Project, exons, which can form mRNA, as well as alternative splicing, receives much more focus than introns, whose meaning is less understood. At the same time, the role played by CNVs is also underestimated, because CNVs are repeated copies with high similarity among copies, and it was only recently that differences in CNVs in different cells from the same individual were unveiled. The confusing functions of Alu elements might also be undervalued given the low expression and short half-lives of these elements. With the mechanism proposed here, the central dogma might be developed in such a way that the genome could be influenced by transcription. We hope that this inspires further study.

## Data Availability Statement

The original contributions presented in the study are included in the article/supplementary material, further inquiries can be directed to the corresponding author/s.

## Author Contributions

YS contributed to the conceptualization, original draft preparation, writing, and drawing, while SP contributed to the revision of the manuscript. All authors have read and agreed to the published version of the manuscript.

## Conflict of Interest

The authors declare that the research was conducted in the absence of any commercial or financial relationships that could be construed as a potential conflict of interest.
